# Effects of persistent modulation of intestinal microbiota on SIV/HIV vaccination in rhesus macaques

**DOI:** 10.1038/s41541-021-00298-4

**Published:** 2021-03-11

**Authors:** Nichole R. Klatt, Courtney Broedlow, Jessica M. Osborn, Andrew T. Gustin, Sandra Dross, Megan A. O’Connor, Ernesto Coronado, Philip Barnette, Tiffany Hensley-McBain, Alexander S. Zevin, Roshell Muir, Alexander Roederer, Solomon Wangari, Naoto Iwayama, Chul Y. Ahrens, Jeremy Smedley, Cassandra Moats, Rebecca M. Lynch, Elias K. Haddad, Nancy L. Haigwood, Deborah H. Fuller, Jennifer A. Manuzak

**Affiliations:** 1 Department of Pharmaceutics, University of Washington, Seattle, WA USA; 2 Washington National Primate Research Center, University of Washington, Seattle, WA USA; 3 Department of Pediatrics, Miller School of Medicine, University of Miami, Miami, FL USA; 4 Division of Surgical Outcomes and Precision Medicine Research, Department of Surgery, University of Minnesota, Minneapolis, MN USA; 5 Department of Microbiology, University of Washington, Seattle, WA USA; 6 Department of Immunology, University of Washington, Seattle, WA USA; 7 Oregon National Primate Research Center, Oregon Health and Science University, Portland, OR USA; 8 Division of Infectious Diseases and HIV Medicine, Drexel University College of Medicine, Philadelphia, PA USA; 9 Department of Microbiology, Immunology and Tropical Medicine, George Washington University School of Medicine and Health Sciences, Washington, DC USA; 10 Tulane National Primate Research Center, Tulane University, Covington, LA USA

**Keywords:** Microbiota, Colon, Rectum, HIV infections, Preclinical research

## Abstract

An effective vaccine to prevent HIV transmission has not yet been achieved. Modulation of the microbiome via probiotic therapy has been suggested to result in enhanced mucosal immunity. Here, we evaluated whether probiotic therapy could improve the immunogenicity and protective efficacy of SIV/HIV vaccination. Rhesus macaques were co-immunized with an SIV/HIV DNA vaccine via particle-mediated epidermal delivery and an HIV protein vaccine administered intramuscularly with Adjuplex™ adjuvant, while receiving daily oral Visbiome^®^ probiotics. Probiotic therapy alone led to reduced frequencies of colonic CCR5^+^ and CCR6^+^ CD4^+^ T cells. Probiotics with SIV/HIV vaccination led to similar reductions in colonic CCR5^+^ CD4^+^ T cell frequencies. SIV/HIV-specific T cell and antibody responses were readily detected in the periphery of vaccinated animals but were not enhanced with probiotic treatment. Combination probiotics and vaccination did not impact rectal SIV/HIV target populations or reduce the rate of heterologous SHIV acquisition during the intrarectal challenge. Finally, post-infection viral kinetics were similar between all groups. Thus, although probiotics were well-tolerated when administered with SIV/HIV vaccination, vaccine-specific responses were not significantly enhanced. Additional work will be necessary to develop more effective strategies of microbiome modulation in order to enhance mucosal vaccine immunogenicity and improve protective immune responses.

## Introduction

With more than 37.9 million people living with HIV globally and 1.7 million new infections per year, HIV remains one of the world’s most devastating infectious diseases^1^, and a vaccine that provides lasting protection against new infections has not been achieved. Although not fully efficacious, the RV144 Thailand HIV vaccine trial resulted in 31.2% efficacy, providing promising evidence that a protective HIV vaccine is attainable^2^. This trial used a combination of the canary-pox vector ALVAC-HIV vCP1521, which expressed clade B Gag-Pro and clade E gp120, in conjunction with gp120 B/E proteins co-formulated in alum adjuvant^2^. At the time of the trial, HIV prevalence in Thailand was 1.7% of the adult population, with 21,260 new infections^3^ and the study participants were at low risk for HIV acquisition. In contrast, in the recent HIV Vaccine Trials Network (HVTN) 702 clinical trial^4^, which built upon RV144, vaccinations were stopped early due to nonefficacy. HVTN 702 utilized ALVAC-HIV vCP2438, which expressed clade B Gag-Pro and clade C gp120, in conjunction with the subtype C gp120 Env proteins, TV1.C and 1806.C, co-formulated with MF59 adjuvant^4^. HVTN 702 was conducted in South Africa, where the HIV prevalence rate in 2018 was 20.4% with >240,000 new infections^1^. The differences in the vaccine populations, administration regimens, and adjuvants likely contributed to the different outcomes observed in RV144 and HVTN 702. Indeed, these results are reflective of a previous study performed in rhesus macaques, which demonstrated that an ALVAC-SIV and gp120 alum vaccine delayed the onset of SIVmac251 infection, while ALVAC-SIV and gp120 MF59 did not reduce the risk of viral acquisition^5^. Taken together, these findings highlight the fact that much work remains to identify an effective HIV vaccination strategy.

Challenges to prophylactic HIV vaccine development include the extreme viral diversity of HIV and an incomplete understanding of the immune correlates of protection^6^. In addition, the complexities of the mucosal immune system present difficulties for the effective induction of cellular and humoral immunity at the mucosal portal of entry^7^. Previous studies have suggested that vaccine platforms utilizing a DNA and protein co-immunization strategy outperform individual or sequential DNA prime and protein boosts, and result in rapid and potent neutralizing antibody and T cell responses capable of inducing immunity against SIV or SHIV challenge in a portion of vaccinated animals^8–13^. In addition, DNA vaccination has been shown to induce SIV-specific responses in the intestinal mucosa^14^. However, the development of a DNA/protein co-immunization strategy that provides complete sterilizing immunity in all vaccinated animals against repeated mucosal SIV or SHIV challenges has not been achieved. Thus, there is a clear need for alternative vaccine strategies that can elicit mucosal responses that provide full protection against SIV/HIV infection.

Manipulation of the intestinal microbiota by probiotic therapy could improve musical immune responses. Previous studies have confirmed that probiotics are well-tolerated by anti-retroviral therapy (ART)-treated HIV-infected individuals, although the overarching conclusions varied between studies^15^. Indeed, several groups demonstrated marked improvement with probiotic therapy, including reduced peripheral and intestinal frequencies of activated CD4^+^ T cells^16,17^ and lower levels of inflammatory markers in the CNS^18^. However, others showed little effect of probiotics on clinically relevant readouts, including systemic inflammatory markers^19^, CD4^+^ T cell counts, and CD4/CD8 ratio^20^. Work using the macaque model demonstrated that prebiotic/probiotic administration in ART-treated SIV-infected macaques resulted in elevated frequencies and functionality of colonic CD4^+^ T cells and antigen-presenting cells^21^ and in combination with IL-21 lead to increased jejunal Th17 cell frequencies and reduced microbial translocation^22^. Notably, probiotic therapy in healthy macaques resulted in decreased frequencies of colonic activated and proliferating CD4^+^ T cells^23^, which are preferential targets of SIV/HIV infection^24^. These findings suggest that probiotics could be used in the context of SIV/HIV vaccine strategies to improve mucosal immune responses without unintentionally increasing mucosal target cells.

Here, we theorized that the immunologic shifts induced by probiotic therapy could simultaneously enhance SIV/HIV vaccine-specific mucosal immunity while limiting the accumulation of potential SHIV cells. To test this hypothesis, we treated rhesus macaques with continuous oral probiotics while concurrently immunizing with a Clade C-based SIV/HIV DNA/protein vaccine regimen; we characterized intestinal microbial communities, mucosal and lymphoid immune populations, and SIV/HIV vaccine immunogenicity and efficacy throughout the study. Although treatment with probiotics produced an immunomodulatory effect, primarily in colonic mucosal tissue, no significant differences in rectal cellular or humoral vaccine responses were observed between Probiotics+Vaccine and Vaccine-only animals prior to intrarectal SHIV challenge. Protection from intrarectal challenge with the heterologous clade C SHIV.CH505 was not observed among any of the vaccinated animals, independent of probiotic treatment. These findings provide an assessment of the ability of an oral probiotic cocktail to modulate mucosal immune cell subsets, enhance SIV/HIV vaccination and prevent mucosal SHIV transmission.

## Results

### Experimental design

**Fig. 1:**
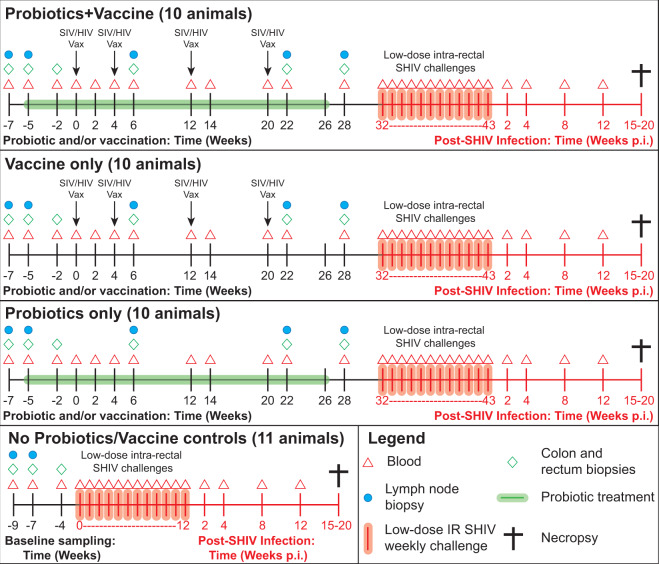
Experimental timeline. Male rhesus macaques were entered into four experimental groups: Probiotics+Vaccine (*n* = 10), Vaccine-only (*n* = 10), Probiotics-only (*n* = 10), and no Probiotics/no Vaccine controls (*n* = 11). For Probiotics+Vaccine and Probiotics-only groups, probiotic treatment began at week −5 and continued through week 26. For Probiotics+Vaccine and Vaccine-only groups, SIV/HIV DNA/protein immunization occurred at weeks 0, 4, 12, and 22. All macaques were intrarectally challenged with SHIV.CH505 and followed for up to 20 weeks post-infection. Collection of samples, including colon, rectum, and lymph node biopsies and whole blood was completed at the specified time points.

Forty-one male, Indian-origin rhesus macaques were split into four groups: (1) Probiotics+Vaccine (*n* = 10), (2) Vaccine-only (*n* = 10), (3) Probiotics-only (*n* = 10), and (4) no Probiotics/no Vaccine control animals (*n* = 11; Fig. 1). From week −5 to 26, animals in the Probiotics+Vaccine and Probiotics-only groups received daily oral Visbiome^®^ probiotics. Animals in the Probiotics+Vaccine and Vaccine-only groups were immunized with an SIV gag (p55) and HIV env (gp160) DNA vaccine administered via particle-mediated epidermal delivery (PMED) and intramuscularly immunized with gp140 trimeric protein at weeks 0, 4, 12, and 20. Four weeks after the last sampling time point (week 28 for experimental groups and week −4 for no Probiotics/no Vaccine control animals), all animals underwent repeated, weekly low-dose intrarectal SHIV.CH505 challenges. Challenges were stopped once an animal tested positive for SHIV infection. Viral kinetics were followed until necropsy between week 15 and 20 post infection. During the probiotics and/or vaccine phase of the study, colon, rectum, jejunum, and peripheral lymph node (LN; inguinal or axillary) biopsy specimens were collected. Peripheral blood was collected at all time points during the probiotics and/or vaccine phase, SHIV challenge, and post-infection monitoring.

### Minimal shifts in microbial taxonomy throughout probiotic administration and vaccination

**Fig. 2:**
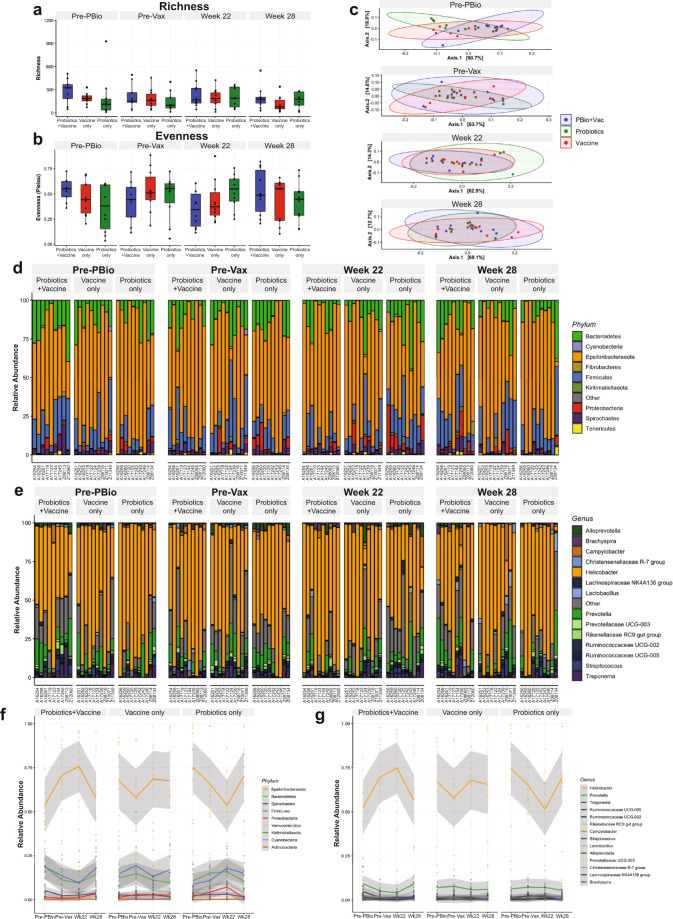
Microbial communities are minimally disrupted in colonic tissue during probiotic administration, SIV/HIV vaccination, or combination Probiotics+Vaccine. 16s rRNA gene sequencing was used to characterize microbial communities in Probiotics+Vaccine (*n* = 10), Vaccine-only (*n* = 10), and Probiotics-only animals (*n* = 10). **a**, **b** Bacterial community richness (**a**) and evenness (**b**). Box and whisker bars represent 25–75 percentile and minimum and maximum number of observed OTUs. Black dots that overlay box and whisker plots represent the total number of observed OTUs for individual animals at each time point. Horizontal bars within each box represent the median. **c** Principal components analysis of beta-diversity. Probiotics+Vaccine animals are shown in blue, Vaccine-only in red, and Probiotics-only in green. Shaded ovals for each group represent data ellipses. **d**, **e** Relative abundance taxonomic plots of microbial phyla (**d**) and genera (**e**). Vertical colored bars represent the percentage of total sequences for specific phyla in individual animals at each of the indicated time points. **f**, **g** Smoothed mean relative abundance of bacterial phyla (**f**) and genera (**g**) at each of the indicated time points. Solid colored lines represent the mean abundance for specific bacterial phyla. Gray shading overlaying each colored line represents standard error bounds. Matched colored dots surrounding each colored line represent the specific abundances of each bacterial phyla or genera for individual animals. Pre-PBio baseline is an average of weeks −7 and −5, while Pre-Vax is week −2.

To begin examining the relationship between probiotic therapy and SIV/HIV vaccination, we used 16s rRNA gene sequencing to profile bacterial communities in the colonic mucosa. No significant differences in community richness (Fig. 2a) or evenness (Fig. 2b) were observed over time in any experimental group. The lack of colonic bacterial community perturbation was further demonstrated by principal components analysis (PCoA), which showed an overlap of experimental groups at all time points (Fig. 2c). In terms of taxonomy, the colonic mucosa of all animals was dominated by bacteria in the Epsilonbacteraeota phyla, with minor representation of Bacteroidetes, Firmicutes, Spirochaetes, Proteobacteria, and Cyanobacteria (Fig. 2d, f and Supplementary Fig. 1). At the genus level, a high abundance of *Helicobacter* was observed in all groups, followed by minor abundances of *Prevotella*, *Treponema*, *Ruminococcaceae*, *Campylobacter*, *Lactobacillus*, *Streptococcus*, and *Alloprevotella* (Fig. 2e, g and Supplementary Fig. 1). These findings are in strong agreement with our and other groups’ assessments of the colonic microbiota of rhesus macaques^25–27^. The Probiotics+Vaccine group experienced a nonsignificant increase in the relative abundance of Epsilonbacteraeota from Pre-PBio to week 22, driven specifically by a significant increase in the relative abundance of genus *Helicobacter* (*P* = 0.0004; Fig. 2d–g and Supplementary Fig. 1). The relative abundances of both Epsilonbacteraeota and *Helicobacter* returned to baseline levels in the Probiotics+Vaccine group by week 28 (Fig. 2d–g and Supplementary Fig. 1). Conversely, the Probiotics-only group exhibited a nonsignificant decrease in the relative abundance of *Helicobacter* and its phylum, Epsilonbacteraeota from Pre-PBio to week 22 which returned to baseline at week 28 (Fig. 2d–g and Supplementary Fig. 1). The abundances of Epsilonbacteraeota and *Helicobacter* fluctuated over time in the Vaccine-only group and illustrate that the changes observed in the Probiotics+Vaccine and Probiotics-only groups may be within the expected variance of these populations (Fig. 2d–g and Supplementary Fig. 1). Finally, no differences in alpha-diversity, beta-diversity, or microbial community abundance were observed between the experimental groups and no Probiotics/no Vaccine controls at week 28 (Supplementary Fig. 2).

### Similar frequency of CD4^+^ T cell subset frequencies in mucosal and LN tissue throughout probiotics, SIV/HIV vaccination, and combination Probiotics+Vaccine

**Fig. 3:**
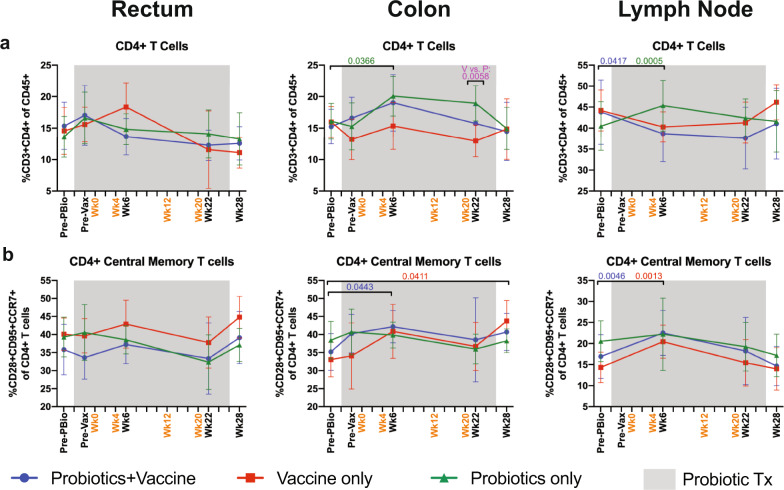
CD4^+^ T cell subset frequencies in mucosal and lymph node tissue during probiotic administration, SIV/HIV vaccination, or combination Probiotics+Vaccine. CD4^+^ T cell subsets were characterized in the rectum, colon, and lymph node of Probiotics+Vaccine (*n* = 10), Vaccine-only (*n* = 10), and Probiotics-only (*n* = 10) treated animals by flow cytometry. **a** Percentage of CD3^+^CD4^+^ T cells of CD45^+^ leukocytes. **b** Percentage of CD4^+^ central memory cells (CD28^+^CD95^+^CCR7^+^) of CD4^+^ T cells. In all panels, data are depicted as the mean and 95% confidence interval for each group: Probiotics+Vaccine = blue circles, Vaccine-only = red squares, Probiotics-only = green triangles. Pre-PBio baseline is an average of weeks -7 and -5, while Pre-Vax is week −2. Immunizations at weeks 0, 4, 12, and 20 are indicated in orange font. Daily oral probiotics were administered between week −5 and week 26, indicated by the gray bar. For comparisons within each group between Pre-PBio and subsequent time points, multiplicity adjusted significant *P* values are shown above horizontal black bars, with fonts colored to indicate the experimental group. For comparisons between groups at each time point, multiplicity adjusted significant *P* values are specified above the designated time point.

Although we did not observe major differences in microbial taxonomy between the three experimental groups, particularly at the last time point prior to the SHIV challenge, differences in microbial functionality due to probiotic therapy could potentially impact mucosal immunity and vaccine responses. Therefore, using our previously described gating strategy^28^, we assessed CD4^+^ T cells (CD3^+^CD4^+^ of CD45^+^ ), including central memory (CD95^+^CD28^+^CCR7^+^ of CD4^+^) and effector memory (CD95^+^CD28^−^CCR7^−^ of CD4^+^) CD4^+^ T cells by flow cytometry (Supplementary Fig. 3). For each cellular subset, comparisons were made at each time point between the three experimental groups and over time within each group compared to the Pre-PBio baseline. CD4^+^ T cell frequencies were significantly increased in the colon and LN of Probiotics-only animals at week 6 compared to the Pre-PBio baseline (*P* = 0.0366 and 0.0005, respectively; Fig. 3a). This elevation likely drove the significant difference in CD4^+^ T cell frequencies between the Probiotics-only and Vaccine-only groups at week 22 in the colon (*P* = 0.0058; Fig. 3a). Conversely, Probiotics+Vaccine animals had a significant decrease in CD4^+^ T cell frequencies in the LN at week 6 (*P* = 0.0417, respectively; Fig. 3a). CD4^+^ central memory T cells were significantly increased in Probiotics+Vaccine animals at week 6 in the colon and LN (*P* = 0.0443 and 0.0046, respectively) and in Vaccine-only animals at week 6 in the LN (*P* = 0.0013) and colon at week 28 (*P* = 0.0411; Fig. 3b). No differences in CD4^+^ effector memory T cells were observed in any experimental group (Supplementary Fig. 4). Finally, to identify potential vaccine-only effects, we also compared the frequency of each cellular subset in the rectum and colon at Pre-Vax with post-vaccine time points and observed that there were no differences in total CD4^+^ T cell frequencies, including central and effector memory subsets, between Pre-Vax and subsequent time points (Supplementary Table 1).

### Significant alterations in the frequency of CCR5^+^ CD4^+^ T cell subsets in mucosal tissue of probiotic-treated animals with or without SIV/HIV vaccination

**Fig. 4:**
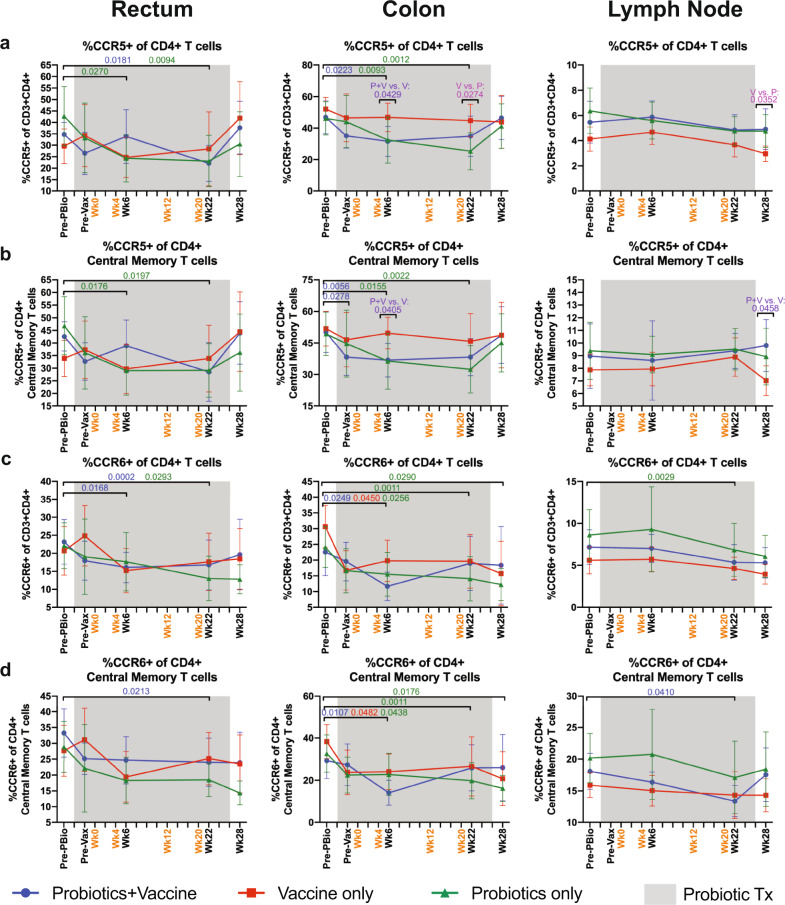
Frequency of CCR5^+^ and CCR6^+^ CD4^+^ T cell subsets in mucosal and lymph node tissue during probiotic administration, SIV/HIV vaccination, or combination Probiotics+Vaccine. CD4^+^ T cell subsets expressing CCR5 or CCR6 were characterized in the rectum, colon, and lymph node of Probiotics+Vaccine (*n* = 10), Vaccine-only (*n* = 10), and Probiotics-only (*n* = 10) treated animals by flow cytometry. **a** Percentage of CCR5^+^ cells of CD4^+^ T cells. **b** Percentage of CCR5^+^ cells of CD4^+^ central memory T cells. **c** Percentage of CCR6^+^ cells of CD4^+^ T cells. **d** Percentage of CCR6^+^ cells of CD4^+^ central memory T cells. In all panels, data are depicted as the mean and 95% confidence interval for each group: Probiotics+Vaccine = blue circles, Vaccine-only = red squares, Probiotics-only = green triangles. Pre-PBio baseline is an average of weeks −7 and −5, while Pre-Vax is week −2. Immunizations at weeks 0, 4, 12, and 20 are indicated in orange font. Daily oral probiotics were administered between week −5 and week 26, indicated by the gray bar. For comparisons within each group between Pre-PBio and subsequent time points, multiplicity adjusted significant *P* values are shown above horizontal black bars, with fonts colored to indicate the experimental group. For comparisons between groups at each time point, multiplicity adjusted significant *P* values are specified above the designated time point.

We next characterized the frequency of CCR5^+^ CD4^+^ T cells in mucosal tissue by flow cytometry. Probiotics+Vaccine animals had significant reductions of CCR5^+^ CD4^+^ Tcells in the rectum at week 22 (*P* = 0.0181) and in the colon at week 6 (*P* = 0.0223) compared to the Pre-PBio baseline (Fig. 4a). Probiotics-only animals had significant reductions of CCR5^+^ CD4^+^ T cells in the rectum and colon by week 6 (*P* = 0.0270 and 0.0093, respectively), which was sustained out to week 22 (*P* = 0.0094 and 0.0012, respectively; Fig. 4a). These reductions likely drove the significant differences in CCR5^+^ CD4^+^ T cell frequencies in the colon between Probiotics+Vaccine and Vaccine-only animals at week 6 (*P* = 0.0429) and between Probiotics-only and Vaccine-only animals at week 22 in the colon (*P* = 0.0274) and week 28 in the lymph node (*P* = 0.0352; Fig. 4a).

In the memory compartment, Probiotics+Vaccine animals had significantly reduced CCR5^+^ CD4^+^ central memory T cells in the colon at Pre-Vax and week 6 (*P* = 0.0278 and 0.0056, respectively; Fig. 4b). Probiotic-only animals had reduced CCR5^+^ CD4^+^ central memory T cells in the rectum and colon at week 6 (*P* = 0.0176 and 0.0155, respectively) and week 22 (*P* = 0.0197 and 0.0022, respectively; Fig. 4b). CCR5^+^ CD4^+^ central memory T cell frequencies were unchanged in Vaccine-only animals, resulting in a significant difference between this group and the Probiotics+Vaccine group at week 6 in the colon (*P* = 0.0405) and week 28 in the lymph node (*P* = 0.0458; Fig. 4b). CCR5^+^ CD4^+^ effector memory cells were significantly reduced in Probiotics-only animals in the rectum and colon at week 22 (*P* = 0.0476 and 0.0479, respectively) and in the LN at week 6 and 22 (*P* = 0.0.0215 and 0.0402, respectively; Supplementary Fig. 4). These cells were significantly reduced in Vaccine-only animals at week 6 compared to Pre-PBio in the LN (*P* = 0.0171; Supplementary Fig. 4) Finally, compared to the Pre-Vax time point, CCR5^+^ CD4^+^ effector memory cells were significantly reduced at week 6 in the rectum of Vaccine-only animals (*P* = 0.0361; Supplementary Table 2).

CCR6^+^ CD4^+^ T cells and proliferating Ki-67^+^ CD4^+^ T cells are targets of SHIV infection^29–32^, thus we characterized the frequency of these cells by flow cytometry. CCR6^+^ CD4^+^ T cells were significantly reduced in Probiotics+Vaccine animals in the rectum at weeks 6 and 22 (*P* = 0.0168 and 0.0002, respectively) and in the colon at week 6 (*P* = 0.0249) as compared to Pre-PBio (Fig. 4c). In addition, CCR6^+^ CD4^+^ T cells were significantly reduced in Probiotics-only animals in the rectum at week 22 (*P* = 0.0293), colon at weeks 6, 22, and 28 (*P* = 0.0256, 0.0011, and 0.0290, respectively), and lymph node at week 22 (*P* = 0.0029) compared to Pre-PBio (Fig. 4c). CCR6^+^ CD4^+^ T cells were significantly reduced in Vaccine-only animals in the colon at week 6 compared to Pre-PBio (*P* = 0.0450; Fig. 4c). Finally, these cells were significantly decreased in the rectum at week 6 compared to Pre-Vax (*P* = 0.0099; Supplementary Table 3).

In the memory compartment, CCR6^+^ CD4^+^ central memory cells were significantly decreased in Probiotics+Vaccine animals the colon at week 6 (*P* = 0.0107) and the rectum and lymph node at week 22 (*P* = 0.0213 and *P* = 0.0410, respectively), as compared to Pre-PBio (Fig. 4d). Probiotics-only animals had decreased CCR6^+^ CD4^+^ central memory cells in the colon at weeks 6, 22, and 28 compared to Pre-PBio (*P* = 0.0438, 0.0011, and 0.0176, respectively; Fig. 4d). Vaccine-only animals exhibited decreased frequencies of these cells in the colon at week 6 compared to Pre-PBio (*P* = 0.0482; Fig. 4d). Compared to Pre-Vax, CCR6^+^ CD4^+^ central memory cells were significantly decreased in the colon of Probiotics+Vaccine animals at week 6 (*P* = 0.0209; Supplementary Table 3). Among effector memory cells, CCR6^+^ expressing CD4^+^ T cells were significantly lower at week 6 compared to Pre-PBio in the colon of Probiotics-only animals (*P* = 0.0046; Supplementary Fig. 4). Finally, only minimal alterations in the frequency of Ki-67^+^ CD4^+^ T cells, including memory subsets were observed over time in the rectum, colon, and lymph node of all three experimental groups (Supplementary Fig. 5 and Supplementary Table 4).

### Minimal disruption in CD8^+^ and Ki-67^+^ CD8^+^ T cell frequencies in mucosal and LN tissue of probiotic-treated animals with or without SIV/HIV vaccination

**Fig. 5:**
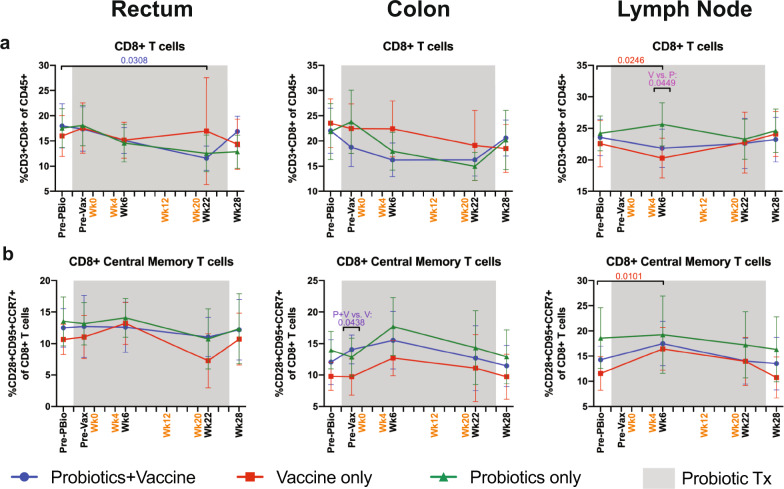
Frequency of CD8^+^ T cell subsets in mucosal and lymph node tissue of during probiotic administration, SIV/HIV vaccination, or combination Probiotics+Vaccine. CD8^+^ T cell subsets were characterized in the rectum, colon, and lymph node of Probiotics+Vaccine (*n* = 10), Vaccine-only (*n* = 10), and Probiotics-only (*n* = 10) treated animals by flow cytometry. **a** Percentage of CD3^+^CD8^+^ T cells of CD45^+^ leukocytes. **b** Percentage of CD8^+^ central memory cells (CD28^+^CD95^+^CCR7^+^) of CD8^+^ T cells. In all panels, data are depicted as the mean and 95% confidence interval for each group: Probiotics+Vaccine = blue circles, Vaccine-only = red squares, Probiotics-only = green triangles. Pre-PBio baseline is an average of week −7 and −5, while Pre-Vax is week −2. Immunizations at weeks 0, 4, 12, and 20 are indicated in orange font. Daily oral probiotics were administered between week −5 and week 26, indicated by the gray bar. For comparisons within each group between Pre-PBio and subsequent time points, multiplicity adjusted significant *P* values are shown above horizontal black bars, with fonts colored to indicate the experimental group. For comparisons between groups at each time point, multiplicity adjusted significant *P* values are specified above the designated time point.

CD8^+^ T cell responses are crucial for vaccine efficacy^33^, thus we assessed the frequency of CD8^+^ T cells, including memory subsets, by flow cytometry. CD8^+^ T cell frequencies were significantly decreased in the rectum in Probiotics+Vaccine group at week 22 compared to Pre-PBio (*P* = 0.0308) and in the Vaccine-only group in the lymph node at week 6 compared to baseline (*P* = 0.0246; Fig. 5a). This reduction drove the significant difference in CD8^+^ T cell frequencies between Vaccine-only and Probiotics-only animals at week 6 in the LN (*P* = 0.0449; Fig. 5a). Compared to Pre-Vax, the Probiotics-only group had reduced CD8^+^ T cell frequencies in the rectum at week 28 (*P* = 0.0336; Supplementary Table 5).

Only subtle differences were observed in the frequency of CD8^+^ memory subsets in the rectum, colon, and LN (Fig. 5b and Supplementary Fig. 6). The Vaccine-only group experienced significant increases in CD8^+^ central memory T cells in the LN at week 6 compared to Pre-PBio (*P* = 0.0101; Fig. 5b). In addition, significantly lower frequencies of these cells were observed in Vaccine-only compared to Probiotics+Vaccine animals at Pre-Vax (*P* = 0.0438; Fig. 5b). Higher colon CD8^+^ effector memory T cell frequencies were observed in Vaccine-only compared to Probiotics+Vaccine animals at week 6 (*P* = 0.0461; Supplementary Fig. 6). Finally, as compared to Pre-Vax, significantly decreased frequencies CD8^+^ effector memory T cells were observed at week 6 in the rectum of Vaccine-only animals and in the colon of Probiotics-only animals (*P* = 0.0271 and 0.0196, respectively; Supplementary Table 5).

To determine whether a lack of cellular proliferation could explain the minimal alterations in CD8^+^ T cells, we assessed the frequency of Ki-67^+^ CD8^+^ T cells. We observed a general trend towards increased percentages, including in memory subsets; however, only the frequency of Ki-67^+^ CD8^+^ central memory T cells became significantly increased in the LN of Probiotics+Vaccine animals at week 22 compared to Pre-PBio (*P* = 0.0479; Supplementary Fig. 6). Compared to Pre-Vax, there was a significant increase in Ki-67^+^ CD8^+^ central memory cells in the rectum of Vaccine-only animals at week 22 (*P* = 0.0340; Supplementary Table 6).

### Reduction of cytokine-producing CD4^+^ T cells but not CD8^+^ T cells in the colon of Probiotics and Probiotics+Vaccine animals

**Fig. 6:**
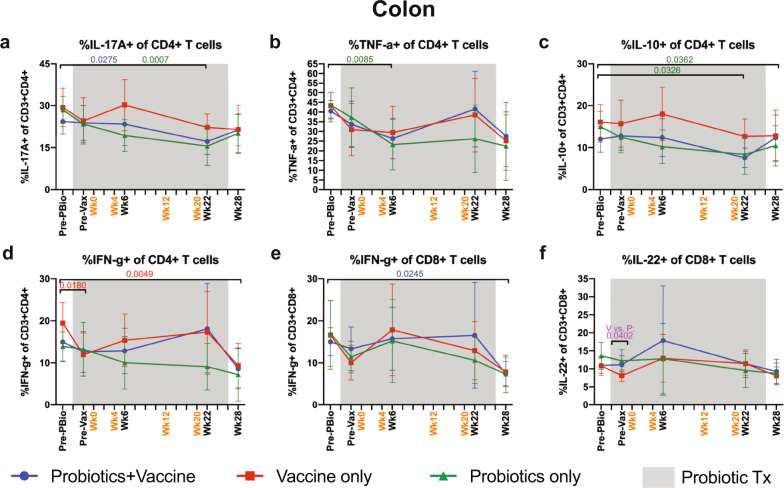
Cytokine-producing CD4^+^ and CD8^+^ T cells in the colon during probiotic administration, SIV/HIV vaccination, or combination Probiotics+Vaccine. CD4^+^ and CD8^+^ T cells producing cytokines were characterized subsequent to mitogenic PMA-ionomycin stimulation in colon tissue of Probiotics+Vaccine (*n* = 10), Vaccine-only (*n* = 10), and Probiotics-only (*n* = 10) treated animals by flow cytometry. **a**–**d** Percentage of CD4^+^ T cells producing IL-17A (**a**), TNF-α (**b**), IL-10 (**c**), and IFN-γ (**d**) in the colon. **e**, **f** Percentage of CD8^+^ T cells producing IFN-γ (**e**) and IL-22 (**f**) in the colon. In all panels, data are depicted as the mean and 95% confidence interval for each group: Probiotics+Vaccine = blue circles, Vaccine-only = red squares, Probiotics-only = green triangles. Pre-PBio baseline is an average of weeks −7 and −5, while Pre-Vax is week −2. Immunizations at weeks 0, 4, 12, and 20 are indicated in orange font. Daily oral probiotics were administered between week −5 and week 26, indicated by the gray bar. For comparisons within each group between Pre-PBio and subsequent time points, multiplicity adjusted significant *P* values are shown above horizontal black bars, with fonts colored to indicate the experimental group. For comparisons between groups at each time point, multiplicity adjusted significant *P* values are specified above the designated time point.

The induction of cytokine-producing T cells is an important antiviral correlate of protection^33^. In addition, production of T cell-derived cytokines such as IL-10, IL-17, and IL-22 are important in maintaining mucosal homeostasis^34^. Thus, we characterized the frequency of cytokine-producing CD4^+^ and CD8^+^ T cells in the colon following mitogenic phorbol myristate acetate (PMA) and ionomycin stimulation by flow cytometry (Fig. 6). Probiotics+Vaccine animals and Probiotics-only animals had a significant decrease in IL-17A^+^ CD4^+^ T cells at week 22 compared to Pre-PBio (*P* = 0.0275 and 0.0007, respectively; Fig. 6a). Probiotics-only animals additionally showed significant decreases in TNF-α^+^ CD4^+^ T cells at week 6 compared to Pre-PBio (*P* = 0.0085; Fig. 6b) and IL-10^+^ CD4^+^ T cells at weeks 22 and 28 compared to Pre-PBio (*P* = 0.0326 and 0.0362, respectively; Fig. 6c). IFN-γ^+^ CD4^+^ T cells were significantly decreased at Pre-Vax and week 28 compared to Pre-PBio in Vaccine-only animals (*P* = 0.0180 and 0.0049, respectively; Fig. 6d). Among CD8^+^ T cells, frequencies of IFN-γ^+^ expressing cells were significantly decreased in Probiotics+Vaccine animals at week 28 compared to Pre-PBio (*P* = 0.0245; Fig. 6e). In addition, a significant difference in IL-22^+^ CD8^+^ T cells was observed between the Vaccine-only and Probiotics-only groups at the Pre-Vax time point (*P* = 0.0402; Fig. 6f). No differences were observed in cytokine-producing CD4^+^ or CD8^+^ T cell frequencies at Pre-Vax as compared to subsequent time points in any group (Supplementary Table 7).

### Elevated SIV Gag-specific CD4^+^ and CD8^+^ T cell responses in Probiotics+Vaccine animals

**Fig. 7:**
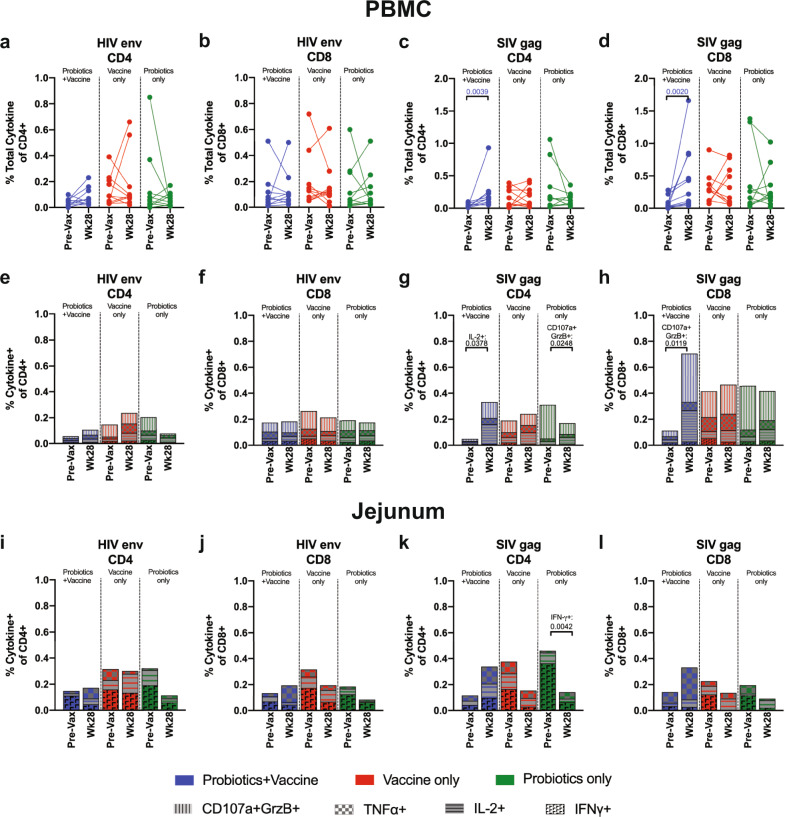
SIV Gag- and HIV Env-specific CD4^+^ and CD8^+^ T cell responses during probiotic administration, SIV/HIV vaccination, or combination Probiotics+Vaccine. SIV- and HIV-specific responses against SIVmac239 Gag or HIV-1 Consensus C Env were characterized in PBMCs and jejunum cells from Probiotics+Vaccine (*n* = 10), Vaccine-only (*n* = 10), and Probiotics-only (*n* = 10) treated animals stimulated with overlapping peptide pools by flow cytometry. Frequencies were considered positive after DMSO-stimulated and baseline subtractions. **a**–**d** Boolean gating was performed to identify the total frequency of CD4^+^ (**a**, **c**) and CD8^+^ (**b**, **d**) T cells within PBMCs with cytokine (IFN-γ, IL-2, and TNF-α) or cytolytic effector (CD107a^+^/granzyme B [GrzB]^+^) functions. Each animal is represented by a filled circle and lines between points connect data from the same animal. (e-h) Mean frequency of CD4^+^ (**e**, **g**) and CD8^+^ (**f**, **h**) T cells within PBMCs with individual cytokine (IFN-γ, IL-2, and TNF-α) or cytolytic effector (CD107a^+^/granzyme B [GrzB]^+^) functions. **i**–**l** Mean frequency of CD4^+^ (**i**, **k**) and CD8^+^ (**j**, **l**) T cells within jejunum cells with individual cytokine (IFN-γ, IL-2, and TNF-α) functions. In all panels, colors indicate different groups: Probiotics+Vaccine = blue; Vaccine-only = red; Probiotics-only = green.

The induction of antigen-specific T cells is an important component of protective immunity and vaccine efficacy^33^. In particular, Env-specific polyfunctional T cell responses were shown to be inversely correlated with HIV infection in the RV144 trial^35^. Thus, we next assessed peptide-specific responses to HIV Env and SIV Gag in peripheral blood mononuclear cells (PBMCs) at week −2 (Pre-Vax) and week 28 by flow cytometry (Fig. 7). No differences in the total CD4^+^ and CD8^+^ T cell cytokine and effector response to stimulation with HIV Env was observed between Pre-Vax and week 28 in the Probiotics+Vaccine, Vaccine-only or Probiotics-only groups (Fig. 7a, b). Increased frequencies of Gag-specific CD4^+^ and CD8^+^ T cells were observed at week 28 compared to Pre-Vax (*P* = 0.0039 and 0.002, respectively), however, no differences were observed in the Vaccine-only or Probiotics-only groups (Fig. 7c, d). Between the three groups, no differences in total CD4^+^ or CD8^+^ T cell responses to HIV Env or CD4^+^ responses to SIV Gag were observed at Pre-Vax or at week 28 (Supplementary Table 8). However, increased frequencies of SIV Gag-specific CD8^+^ T cells were observed in the Vaccine-only group compared to the Probiotics+Vaccine group (*P* = 0.0063; Supplementary Table 8).

To assess individual effector functions, we characterized the frequency of PBMC CD4^+^ or CD8^+^ T cells producing IFN-γ, IL-2, TNF-α, or CD107a/Granzyme B (GrzB) in response to HIV Env or SIV Gag. No differences in individual CD4^+^ or CD8^+^ T cell responses to HIV Env were observed between Pre-Vax and week 28 in any of the experimental groups (Fig. 7e, f). A significant increase in Gag-specific IL-2^+^ CD4^+^ T cells (*P* = 0.0378) and CD107a^+^GrzB^+^ CD8^+^ T cells (*P* = 0.0119) were observed in the Probiotics+Vaccine group at week 28 compared to Pre-Vax (Fig. 7g, h). Gag-specific CD107a^+^GrzB^+^ CD4^+^ T cells were decreased at week 28 compared to Pre-Vax in the Probiotics-only group (*P* = 0.0248; Fig. 7g). Between the three groups, the frequency of CD107a^+^GrzB^+^ HIV Env-specific CD4^+^ T cells in the Probiotics-only group was significantly higher compared to Probiotics+Vaccine at Pre-Vax (*P* = 0.0388), but significantly lower than the Vaccine-only group at week 28 (*P* = 0.0351; Supplementary Table 9). In response to SIV Gag, the frequency of CD107a^+^GrzB^+^ CD4^+^ T cells was significantly higher at Pre-Vax in the Probiotics-only group as compared to Probiotics+Vaccine (*P* < 0.0001) and Vaccine-only (*P* = 0.0054; Supplementary Table 9). Finally, the frequency of CD107a^+^GrzB^+^ SIV Gag-specific CD8^+^ T cells were significantly lower in the Probiotics+Vaccine group compared to Probiotics-only at Pre-Vax (*P* = 0.0003; Supplementary Table 9).

We further assessed individual effector functions in mucosal tissue by characterizing the frequency of CD4^+^ or CD8^+^ T cells in the jejunum producing IFN-γ, IL-2, or TNF-α in response to HIV Env or SIV Gag. No differences in the CD4^+^ or CD8^+^ T cell response to HIV Env was observed between Pre-Vax and week 28 in each of the three experimental groups in the jejunum (Fig. 7i, j). The frequency of IFN-γ^+^ SIV Gag-specific CD4^+^ T cells was significantly decreased at week 28 compared to Pre-Vax in the jejunum in the Probiotics-only group (*P* = 0.0042, Fig. 7k). No differences in CD8^+^ T cell responses to SIV Gag between Pre-Vax and week 28 were observed in the jejunum (Fig. 7l). Between the three groups, the frequency of Gag-specific IFN-γ^+^ CD4^+^ T cells was significantly higher at Pre-Vax in the Probiotics-only group compared to the Probiotics+Vaccine group (*P* = 0.0015; Supplementary Table 9). Finally, the frequency of Gag-specific TNF-α^+^ CD8^+^ T cells was significantly higher at week 28 in the Probiotics+Vaccine group compared to Vaccine-only (*P* = 0.0157) and Probiotics-only (*P* = 0.0032; Supplementary Table 9).

### Increased B cell frequencies in mucosal tissue of probiotic-treated animals and development of humoral immune response in vaccinated animals

**Fig. 8:**
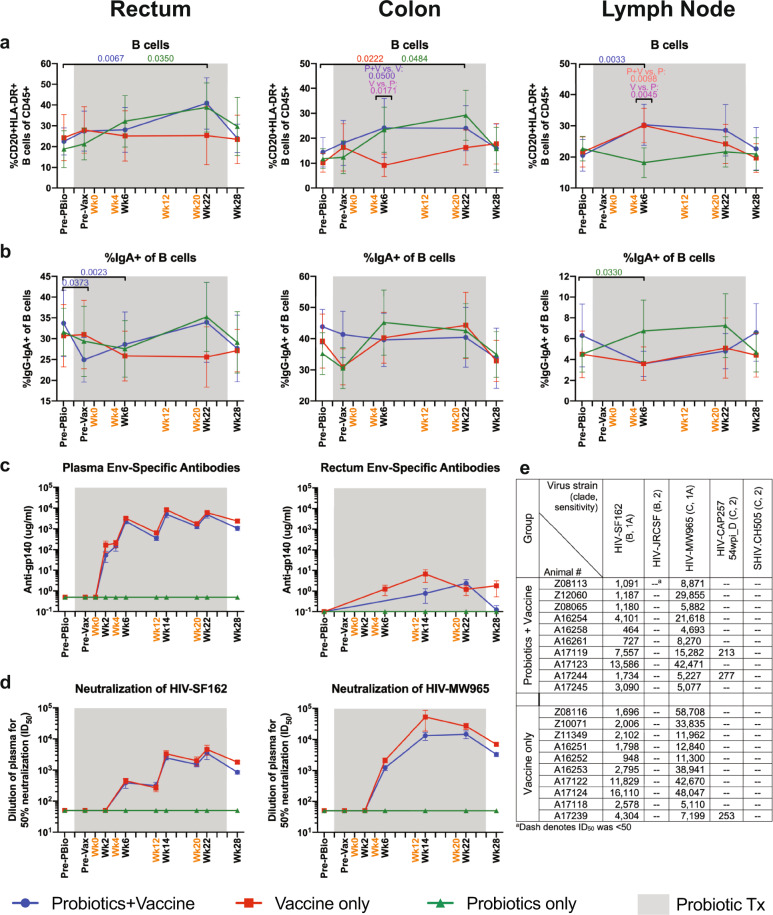
B cell frequencies in mucosal and lymph node tissue and humoral immune responses during probiotic administration, SIV/HIV vaccination, or combination Probiotics+Vaccine. Total CD20^+^HLA-DR^+^ B cells and IgA^+^ B cell frequencies were characterized in the rectum, colon, and lymph node of Probiotics+Vaccine (*n* = 10), Vaccine-only (*n* = 10), and Probiotics-only (*n* = 10) treated animals by flow cytometry. **a** Percentage of CD20^+^HLA-DR^+^ B cells of CD45^+^ leukocytes. **b** Percentage of IgA^+^ of B cells. **c** Mean plasma antibody titers (µg/ml) of Env-specific (gp140) binding antibodies in plasma and rectal secretions. **d** Dilution of plasma required to reach 50% neutralization (ID_50_) of HIV-SF162 and HIV-MW965. **e** Table of neutralization (ID_50_) of HIV-SF162, HIV-JRCSF, HIV-MW965, HIV-CAP257 54wpi_D, and SHIV.CH505 in Probiotics+Vaccine and Vaccine-only animals at peak response (week 22). In all panels, data are depicted as the mean and 95% confidence interval for each group: Probiotics+Vaccine = blue circles, Vaccine-only = red squares, Probiotics-only = green triangles. Pre-PBio baseline is an average of weeks −7 and −5, while Pre-Vax is week −2. Immunizations at weeks 0, 4, 12, and 20 are indicated in orange font. Daily oral probiotics were administered between week −5 and week 26, indicated by the gray bar. For comparisons within each group between Pre-PBio and subsequent time points, multiplicity adjusted significant *P* values are shown above horizontal black bars, with fonts colored to indicate the experimental group. For comparisons between groups at each time point, multiplicity adjusted significant *P* values are specified above the designated time point.

We next assessed the kinetics of B cell frequencies, including IgA^+^ and IgG^+^ B cells by flow cytometry. CD20^+^HLA-DR^+^ B cells were significantly increased in Probiotics+Vaccine animals at week 22 in the rectum (*P* = 0.0067) and week 6 in the lymph node (*P* = 0.0033; Fig. 8a). In addition, these cells were significantly increased in Probiotics-only animals at week 22 compared to Pre-PBio in the rectum and colon (*P* = 0.0350 and 0.0484, respectively; Fig. 8a). B cell frequencies were increased in the colon of Vaccine-only animals at week 22 compared to Pre-PBio (*P* = 0.0222 Fig. 8a). Significant differences were observed at week 6 between the Vaccine-only group and the Probiotics+Vaccine group (*P* = 0.05) and the Probiotics-only group (*P* = 0.0171; Fig. 8a). In addition, significant differences were observed between Probiotics-only animals and the Probiotics+Vaccine group (*P* = 0.0098) and the Vaccine-only group (*P* = 0.0045) in the LN at week 6 (Fig. 8a). Finally, as compared to Pre-Vax, the frequency of B cells was significantly increased in the rectum of Probiotics-only animals at week 22 (*P* = 0.0229; Supplementary Table 10).

IgA^+^ B cells were significantly decreased in Probiotics+Vaccine animals in the rectum at Pre-Vax and week 6 compared to Pre-PBio (*P* = 0.0373 and 0.0023, respectively; Fig. 8b). Conversely, IgA^+^ B cells were significantly increased in the LN of Probiotics-only animals at weeks 6 (*P* = 0.0330; Fig. 8b). No alterations in IgG^+^ B cell frequencies were observed in any of the experimental groups over time (Supplementary Fig. 7 and Supplementary Table 10).

Immune correlates analysis in the RV144 trial demonstrated that high Env-specific binding antibody titers were associated with vaccine efficacy^36,37^. Thus, we assessed production of Clade C Env-specific binding antibodies in plasma and rectal mucosal secretions (Fig. 8c). Env-specific antibodies were detected in Probiotics+Vaccine and Vaccine-only animals in plasma by week 2 after vaccination and were statistically significant compared to Pre-PBio by week 6 in the Probiotics+Vaccine and Vaccine-only group (Fig. 8c). Binding antibodies increased in plasma with each immunization in the two vaccine groups. Env-specific antibodies were detected in Probiotics+Vaccine and Vaccine-only animals in rectal secretions at weeks 14 and 22 (Fig. 8c). Binding antibodies remained detectable in rectal secretions in the Vaccine-only group through week 28, while levels in the Probiotics+Vaccine group decreased to baseline (Fig. 8c). Env-specific antibodies in plasma and rectal secretions were not detected in Probiotics-only animals at any time point (Fig. 8c).

The induction of broadly neutralizing antibody responses is a critical goal for HIV vaccine development^38^. Thus, we assessed neutralizing antibodies in plasma against a panel of viruses, including HIV-SF162 (subtype B, Tier 1a), HIV-JRCSF (subtype B, Tier 2), HIV-MW965 (subtype C, Tier 1a), HIV-CAP257 54wpi_D (subtype C, Tier 2), and SHIV.CH505 (subtype C, Tier 2; challenge virus). Neutralizing antibodies against HIV-SF162 were readily detected in the Probiotics+Vaccine and Vaccine-only group starting at week 6 (Fig. 8d). Neutralizing titers were not augmented by the final immunization at week 20 and were similar at weeks 14, 22, and 28 within the Probiotics+Vaccine and Vaccine-only groups. As with HIV-SF162, neutralizing antibodies against HIV-MW965 were detected at week 6 and were similar at weeks 14, 22, and 28 within the Probiotics+Vaccine and Vaccine-only groups (Fig. 8d). At the time of peak antibody responses (week 22), low-neutralizing antibody responses against HIV-CAP257 54wpi_D were detected in two Probiotics+Vaccine animals and one Vaccine-only animal (Fig. 8e). Significant neutralization of the Tier 2 viruses HIV-JRCSF and SHIV.CH505 has not observed in any of the Probiotics+Vaccine and Vaccine-only animals at week 22 (Fig. 8e). Finally, neutralizing antibodies were not detected in Probiotics-only animals against any of the viruses tested at any time point.

### Similar rate of SHIV.CH505 acquisition, post-infection viral kinetics and peripheral CD4^+^ T cell counts between probiotic-treated, SIV/HIV vaccinated, and Probiotics+Vaccine combination

**Fig. 9:**
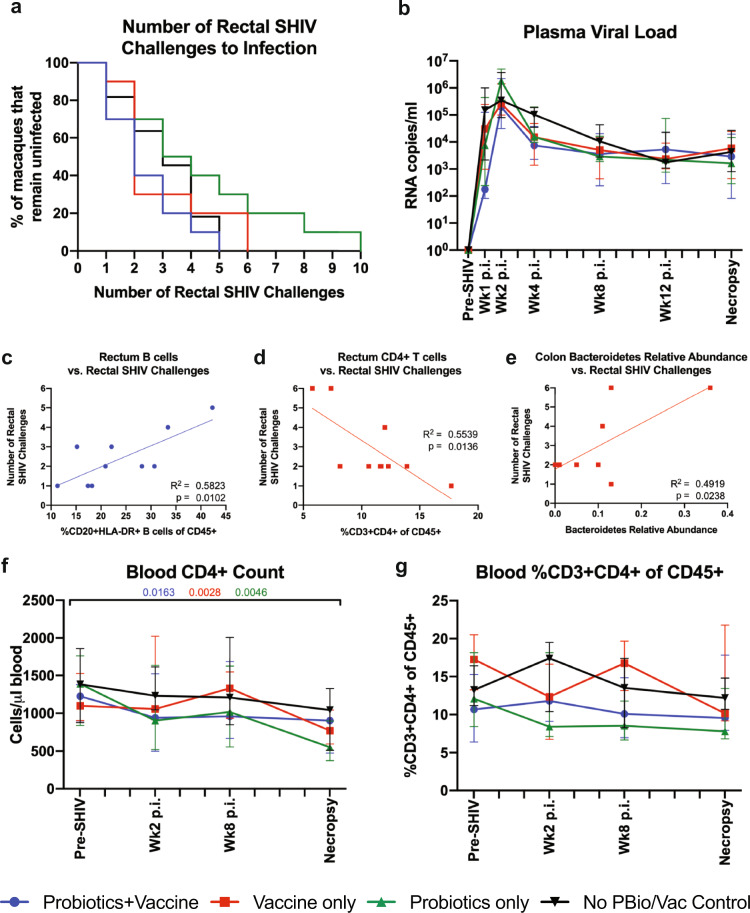
SHIV.CH505 acquisition, post-infection viral kinetics, and peripheral CD4^+^ T cell counts between probiotic-treated, SIV/HIV vaccinated, and combination Probiotics+Vaccine-treated animals. SHIV.CH505 infectivity rate, post-infection (p.i.) viral kinetics, and blood CD4^+^ T cell count and frequency were assessed in Probiotics+Vaccine (*n* = 10), Vaccine-only (*n* = 10), Probiotics-only (*n* = 10), and no Probiotics/no Vaccine controls (*n* = 11). **a** Survival curve showing the percentage of animals that remained uninfected after each rectal challenge. **b** Plasma viral loads (RNA copies/ml plasma). **c**, **d** Linear regressions between the rate of SHIV acquisition and the frequency of B cells in the rectum of Probiotics+Vaccine animals (**c**), the frequency of CD4^+^ T cells in the rectum of Vaccine-only animals (**d**), and the relative abundance of the Bacteroidetes phylum in the colon of Vaccine-only animals (**e**). **f** Absolute number of CD4^+^ T cells per µl of blood. **g** Percentage of CD3^+^CD4^+^ T cells of CD45^+^ leukocytes in whole blood. In all panels, colors indicate different groups: Probiotics+Vaccine = blue circles, Vaccine-only = red squares, Probiotics-only = green triangles. In **b**, **f**, and **g**, data are depicted as the mean and 95% confidence interval for each group. Data at Pre-SHIV time point is week 28 for Probiotics+Vaccine, Vaccine-only, and Probiotics-only, and an average of weeks −9, −7, and −4 for no Probiotics/no Vaccine control animals. For comparisons within each group between Pre-SHIV and subsequent time points, multiplicity adjusted significant *P* values are shown above horizontal black bars, with fonts colored to indicate the experimental group. *R*^2^ and *P* values are shown for each linear regression performed.

To examine whether probiotic treatment enhanced SIV/HIV vaccine efficacy, we carried out a repeated, titered intrarectal SHIV.CH505 challenge. Although from the same clade, this challenge virus was heterologous to the vaccine and is a Tier 2 virus based on neutralization; the vaccine regimen did not induce neutralizing antibodies against CH505 virus (Fig. 8e). All animals became SHIV infected within ten challenges, with an overall 37.9% infectivity rate (Fig. 9a). When divided by group, Probiotics+Vaccine, Vaccine-only, Probiotics-only, and control animals exhibited infectivity rates of 54.6%, 34.9%, 28.7%, and 45.76%, respectively (Fig. 9a). No significant differences were detected in the rate of acquisition between the control group and the Probiotics+Vaccine, Vaccine-only, and Probiotics-only groups (*P* = 0.2861, 0.7637, and 0.2138, respectively; Fig. 9a).

After initial infection, the median peak viral load was reached by 2 weeks post-infection with no significant differences between any of the groups (Fig. 9b). Notably, one animal in the Probiotics+Vaccine group initially exhibited detectable virus at week 1 post-infection, but by week 2 post-infection had no detectable viral load and remained negative for the duration of the study (Supplementary Fig. 8). This animal did not possess protective Mamu-A*01, Mamu-B*08, or Mamu-B*17 alleles that have been associated with improved control of SIV infection (Supplementary Table 11) and were similar in immune phenotype, vaccine-specific responses, and rate of SHIV acquisition similar to other animals. Additional work will be needed to understand why this animal was able to control the virus shortly after the initial infection.

To characterize whether specific immune responses or bacterial communities were associated with virus acquisition, we performed linear regressions between the number of SHIV challenges to infection with mucosal immune responses and microbial communities in each experimental group (Fig. 9c–e). We observed that the frequency of B cells in the rectum of Probiotics+Vaccine animals was positively associated with the number of SHIV challenges (*R*^2^ = 0.5823, *P* = 0.0102; Fig. 9c). In addition, the frequency of CD4^+^ T cells in the rectum of Vaccine-only animals was negatively associated with the number of SHIV challenges (*R*^2^ = 0.5539, *P* = 0.0136; Fig. 9d). Lastly, the relative abundance of the Bacteroidetes phyla was positively associated with the number of SHIV challenges in the Vaccine-only group (*R*^2^ = 0.4919, *P* = 0.0238; Fig. 9e).

Finally, we assessed the kinetics of CD4^+^ T cell loss after SHIV.CH505 infection by flow cytometry (Fig. 9f, g). Consistent with our previous findings^28^, the absolute number of blood CD3^+^CD4^+^ T cells showed moderate declines over time after SHIV infection, with significant decreases by the necropsy time point in the Probiotics+Vaccine group (*P* = 0.0163), the Vaccine-only group (*P* = 0.0028), and the Probiotics-only group (*P* = 0.0046; Fig. 9f). No significant changes were observed in the percentage of blood CD3^+^CD4^+^ T cells over time after SHIV infection (Fig. 9g).

## Discussion

The overall goal of this study was to comprehensively characterize the impact of continuous oral probiotic therapy on the immunogenicity and efficacy of an SIV/HIV DNA/protein co-immunization strategy. Our data indicate that although this particular vaccine strategy elicited both T and B cell adaptive responses in vivo in rhesus macaques, it did not provide protection against the stringent, heterologous, intrarectal SHIV.CH505 challenge. The choice of Env used here was based on its antigenicity and immunogenicity as a DNA/protein vaccine in rabbits and in macaques^39^. In addition, our findings are in line with recent work demonstrating that rhesus macaques vaccinated with this Env in a similar DNA/protein co-immunization formulation also elicited vaccine-specific cellular and humoral immune responses, but were unable to protect against a rigorous heterologous repeated titered intrarectal challenge with a Tier 2 clade C SHIV (SHIV-1157ipd3N4)^40^. The development of vaccines that can elicit robust neutralizing antibodies effective against heterologous Tier 2 viruses remains an elusive target, as Tier 2 neutralization correlates with protection in homologous SHIV challenge studies^41^. Improvements to future vaccines could include the use of a more immunogenic Env and/or the development of an optimized glycoprotein trimer whose antigenic properties more closely resemble native Env on the HIV virion^41^. Also, sustained slow release of glycoprotein immunogens has shown some promise in rhesus macaques to enhance immunity^42^. Finally, co-immunization of DNA and protein vaccines into the same anatomical site was recently shown to induce higher Env-specific humoral and cellular immune responses and greater protection against SHIV.CH505 acquisition^10^. Here, although the DNA vaccine was administered to each inner thigh and protein immunization to one outer thigh, it is possible that even this separation may have impacted the induction of robust immunity. Taken together, future studies to assess the ability of more immunogenic Envs in combination with improved routes of DNA/protein co-administration are warranted.

Although probiotic therapy elicited immunological alterations in both intestinal and lymphoid tissue, probiotic administration was halted after the vaccinations were complete and the majority of the observed immune alterations returned to baseline levels by the last time point prior to the SHIV challenge (week 28). Moreover, significant differences in microbial communities were not observed between the experimental groups prior to the intrarectal SHIV challenge. Together, these data provide a potential explanation for why we did not observe probiotic-enhanced vaccine-specific responses or improved protection against infection. The lack of probiotic integration into the pre-existing commensal microbiome was unsurprising, as previous work has suggested that there may be individual-specific resistance to probiotic colonization^43^. Instead, shifts in microbial-derived metabolites from probiotic bacteria could be the main drivers behind modulated immunity. For example, microbial fermentation of metabolic products and production of short-chain fatty acids (SCFAs) are important in regulating immunity and inflammation^44,45^. Additional work will be needed to quantify microbial metabolites throughout probiotic treatment and determine their association with vaccine-specific responses. Furthermore, because different probiotic formulations may elicit varying immune responses, alternative microbial cocktails designed to induce specific immune responses could promote an optimal mucosal environment for distinct vaccine strategies. Indeed, we observed that the rate of SHIV acquisition was associated with the relative abundance of Bacteroidetes in Vaccine-only animals, however, the Visbiome^®^ probiotics used in this study did not include species in this phylum. Thus, future investigations are needed to determine whether more targeted manipulation of species within the Bacteroidetes phylum could promote vaccine protection against SHIV infection. Finally, given that our data suggest that the immunomodulatory effects of probiotics fade after halting administration, future studies should focus on developing methods to extend microbial-derived effects without the need for long-term daily treatment.

Our data indicate that central memory CD4^+^ and CD8^+^ T cells were significantly, but transiently, increased in colonic and lymph node tissue of vaccinated animals. Conversely, CD4^+^ and CD8^+^ effector memory T cells were unchanged throughout the study. Vaccine-specific memory T cell responses are critical in protection against SIV/HIV infection^46–48^, thus our findings could be indicative of moderate immune response to SIV/HIV vaccination. Moreover, the lack of a sustained and robust central memory response, particularly in the rectum, which was the site of viral exposure, may explain why protection from SHIV acquisition was not observed. Further studies to determine why our vaccine regimen did not induce a strong memory T cell response could help to guide future investigations into novel vaccination strategies.

Limiting the expansion of preferential target cells, including CCR5^+^ or CCR6^+^ expressing CD4^+^ T cells, is of critical importance in rational HIV vaccine design^49,50^. Although probiotic therapy was associated with significant reductions in mucosal CCR5^+^ and CCR6^+^ CD4^+^ T cells, this decrease did not result in a slower rate of SHIV acquisition. Given that probiotic administration was halted prior to viral exposure, a possible explanation for our observations could be that the effect of probiotics on SHIV target populations was lost by the time of the SHIV challenge. Indeed, we did not observe significant differences in these cell subsets in the rectum between any of the experimental groups at the final time point prior to SHIV challenge. Follow-up studies should explore whether altering the timing of vaccination and microbiome manipulation, such as immunization first with subsequent microbial adjustments, could enhance protection against SIV/HIV acquisition.

Previous investigations have suggested that Gag-specific CD4^+^ and CD8^+^ T cell responses were associated with viral control in acute HIV infection^51,52^. In addition, data from the STEP HIV vaccine study suggested that the breadth of the Gag-specific immune response was associated with increased control of viral replication after HIV acquisition^53^, however, the trial failed to prevent acquisition. Here, all experimental groups had predominantly SIV Gag-specific CD4^+^ and CD8^+^ T cell responses and although these responses were enhanced in the Probiotics+Vaccine group, there was no evidence that they contributed to virus control after the acquisition. Moreover, the lack of robust differences between the three experimental groups in vaccine-specific T cell responses in the periphery and mucosa at week 28 could provide an explanation for the absence of differences in SHIV acquisition. Further work will be needed to determine whether pairing manipulation of microbial communities with a more effective mucosal vaccine that elicits efficient T cell responses would result in significant enhancement of vaccine efficacy.

Our data indicate that Probiotics+Vaccine animals did not produce levels of Env-specific binding antibodies in plasma or rectal secretions or neutralizing antibody responses greater than the Vaccine-only condition, providing an additional explanation for why protection against infection was not different between these groups. Surprisingly, in contrast to our previous work^23^, probiotic therapy did not result in expanded IgA^+^ B cells in mucosal tissue. Immune correlates analysis in the RV144 trial demonstrated that high serum Env-specific IgA levels were positively associated with increased risk of HIV acquisition^54^, while macaque studies have revealed that vaccine-induced Env-specific rectal IgA levels were associated with reduced rates of SIV acquisition^55^. More work will be needed to evaluate whether an alternative means of microbiome manipulation would be better suited to enhance the appropriate vaccine-specific mucosal B cell responses needed for protection against infection.

We observed that the frequency of B cells in the rectum of Probiotics+Vaccine animals was positively correlated with the number of SHIV challenges. Interestingly, previous work has demonstrated that Env-specific rectal B cells were associated with delayed SIV acquisition in vaccinated females, but not male macaques^56^. A limitation of our study was that in an effort to constrain the inherent variability in mixed-sex experimental groups, we restricted our study to male macaques. However, given that sex is an important biological factor, both in terms of the demonstrated sex-specific differences in vaccine responses and the high prevalence of HIV among females in endemic areas^57,58^, future studies to characterize potential sex-specific impacts of probiotic administration on SIV/HIV vaccination are necessary.

In summary, our data indicate that although probiotic administration elicited immunological alterations, particularly in the colon, these changes did not result in enhanced immunogenicity of this particular SIV/HIV vaccine platform, nor in greater protection against SHIV acquisition after intrarectal challenge. Modulating the microbiome to skew immune responses has potential clinical implications, as the application of microbial interventions could allow for enhanced mucosal vaccine efficacy. We cannot discount that microbiome modulation may be effective in enhancing responses to a more immunogenic and effective vaccine than the one investigated in this study. Future studies to characterize the impact of alternative and more effective methods of microbial manipulation on vaccine efficacy should be completed in the context of protective SIV/HIV vaccine strategies. Taken together, as progress toward an efficacious HIV vaccine continues, our work highlights the need to continue exploring alternative strategies to improve mucosal vaccine responses and enhance protection against HIV transmission.

## Methods

### Study animals and approval

Forty-one male Indian-origin rhesus macaques (*Macaca mulatta*) were utilized in this study. Expression of the protective Mamu-A*01, Mamu-B*08, and Mamu-B*17 alleles for each animal are detailed in Supplementary Table 11. All animals were housed and cared for at the Washington National Primate Research Center (WaNPRC) under a protocol that was reviewed and approved by the University of Washington Office of Animal Welfare (OWA) Institutional Animal Care and Use Committee (IACUC; Protocol 4266-13; Animal Welfare Assurance Number D16-00292). Animal housing, care, and procedures were performed in an AAALAC-accredited facility, in accordance with the regulations put forth by the United States Department of Agriculture, including the Animal Welfare Act (9 CFR) and the Animal Care Policy Manual and with the guidelines established by the National Research Council in the Guide for the Care and Use of Laboratory Animals and the Weatherall Report. Animals were housed in stainless steel cages with a 12/12 light cycle. One week prior to SHIV challenge, animals in full social contact were shifted into protected (grooming) contact only, in order to limit the unintended exchange of the virus between serodiscordant social partners. Full social contact resumed when all partners tested SHIV-positive. All cage pans and animal rooms were cleaned daily and sanitized at least once every 2 weeks. Animals were provided with a commercial primate chow (Lab Diet, PMI Nutrition International) twice a day, with daily fruits and vegetables and water ad libitum. Environmental enrichment consisted of novel food items, foraging opportunities, and destructible and indestructible manipulanda. For minor procedures (blood collection, SIV/HIV vaccination, SHIV challenges), animals were anesthetized with ketamine (10 mg/kg) and dexmedetomidine (0.017 mg/kg). For more involved sample collection, (colon, rectum, and lymph node biopsies) general anesthesia was maintained with isoflurane by inhalation and postoperative analgesia was provided. Euthanasia was performed via an IV overdose (>75 mg/kg) of pentobarbital in accordance with the recommendations in the Guidelines for the Euthanasia of Animals set forth by the panel on Euthanasia of the American Veterinary Medical Association (AVMA).

### Probiotic administration

Twenty rhesus macaques with (*n* = 10) or without (*n* = 10) concurrent SIV/HIV DNA/protein vaccination received commercially available Visbiome^®^ probiotics (ExeGi Pharma, Rockville, MD) daily starting at Week-5 through Week26. The Visbiome^®^ probiotic cocktail consists of eight bacterial strains, including *Lactobacillus paracasei*, *Lactobacillus plantarum*, *Lactobacillus acidophilus*, *Lactobacillus helveticus*, *Bifidobacterium lactis*, *Bifidobacterium infantus*, *Bifidobacterium breve*, and *Streptococcus thermophilus*. Up to 675 billion bacteria (6 capsules; 112.5 billion bacteria/capsule) were mixed with food and provided to each animal. Macaques were observed by animal staff to ensure that all probiotic food mixtures were consumed. No adverse events due to probiotic therapy were observed in any of the animals.

### Vaccine regimen

Twenty rhesus macaques were immunized with a vaccine platform consisting of a SIV/HIV DNA vaccine co-administered with HIV gp140 protein trimer at Week 0, 4, 12, and 20 with (*n* = 10) or without (*n* = 10) probiotic administration delivered orally. The DNA vaccine consisted of SIV gag (p55) and HIV env (gp160) plasmids, co-formulated with a plasmid expressing the bacterial toxin heat-labile enterotoxin (LT) at a 1:10 ratio, to increase mucosal and systemic immunogenicity^59,60^. Plasmids were precipitated onto separate gold particles as previously described^60–62^. Particle-mediated epidermal delivery (PMED) via the PowderJect^®^ XR1 gene delivery device (PowderJect Vaccines, Inc., Middleton, WI) was used to administer 16 µg of the DNA vaccine into eight skin targets over the inguinal lymph nodes, with the right and left inner thighs each receiving four inoculations. Skin sites were clipped of fur and swabbed with alcohol prior to PMED administration.

Concurrent to PMED DNA administration, animals were co-immunized with CAP257 54wpi_D trimeric gp140 protein. This uncleaved protein trimer is identical to the gp160 expressing DNA used in the DNA vaccine and is 75% identical to the Env in the SHIV challenge stock at the amino acid level. The antigenicity of both protein and DNA have been described previously^39,40^. To prepare the trimeric protein, gp140 DNA was derived from the gp160 envelope 54wk_D sequence by site-directed mutagenesis (QuickChange Multi-Site-Directed Mutagenesis Kit, Stratagene, La Jolla, CA) to insert the previously described mutations^63,64^ in the primary and secondary protease cleavage sites, respectively; REKR was mutated to RSKS and KAKRR was mutated to KAISS. A large-scale endotoxin-free plasmid preparation (Qiagen, Valencia, CA) was used for stable expression in 293F cells for protein production^65^. The protein immunization was co-delivered by administering one 500 µl intramuscular injection into the outer thigh containing either 50 mg (weeks 0 and 4) or 100 µg (weeks 12 and 20) of gp140 trimeric protein. Protein immunization was formulated either in 20% Adjuplex Vaccine Adjuvant (Sigma-Aldrich, St. Louis, MO), which is a purified lecithin and carbomer homopolymer matrix^66^, as has been used previously^67,68^ or 50% AddaVax (InvivoGen, San Diego, CA), a squalene-based oil-in-water nano-emulsion, supplemented with 100ug/ml of Phosphorylated HexaAcyl Disaccharide (PHAD) TLR4 agonist (Avanti Polar Lipids, Alabaster, AL).

### Sample collection and tissue processing

Rectum, colon, and lymph node biopsies, and peripheral blood were collected and processed at specified time points as previously described^28^. Briefly, to collect colon and rectum biopsies, an endoscope or speculum was inserted into the rectum, and biopsies were taken using sterile biopsy forceps. One biopsy was immediately flash-frozen at −80 °C for 16s rRNA gene analysis. Remaining biopsies were enzymatically digested and ground through a 70-µM cell strainer into a single-cell suspension in R10 medium (RPMI 1640 medium with 2.05 nM L-glutamate, supplemented with 10% fetal bovine serum (FBS), 100 U/ml penicillin, and 100 µg/ml streptomycin (all from GE Healthcare)), as previously described^23,28^. Biopsy cell suspensions were immediately stained for flow cytometric analysis.

Inguinal or axillary lymph node biopsies were collected via surgical removal, then ground through a 70 µM cell strainer into a single-cell suspension in R10 medium, as previously described^23,28^. LN cell suspensions were either stained immediately for flow cytometric analysis or were cryopreserved in freezing medium (FBS with 10% dimethyl sulfoxide (DMSO; Sigma-Aldrich)) and stored in liquid nitrogen for future analysis.

Vacutainer blood collection tubes with EDTA anticoagulant (BD, Franklin Lakes, NJ) were used to collect peripheral blood via venipuncture. Whole blood was centrifuged at 2000 r.p.m. for 10 min at 22 °C to separate cells and plasma, and plasma was removed and stored at −80 °C for future analysis. Peripheral blood mononuclear cells were isolated from remaining whole blood by density-gradient centrifugation using Histopaque and Accuspin tubes (both from Sigma-Aldrich). PBMCs were either stained immediately for flow cytometric analysis or were cryopreserved in a freezing medium and stored in liquid nitrogen for future analysis. Complete blood counts with differential were measured on a Beckman Coulter AC*T 5diff CP hematology analyzer (Beckman Coulter, Brea, CA).

### SHIV challenge and viral load quantification

SHIV challenges of all rhesus macaques in this study were performed as detailed previously^28^. Briefly, once per week, animals were intrarectally inoculated with 1 ml of SHIV.CH505.375H.dCT^69^ (SHIV.CH505) diluted 1:100 in RPMI 1650 medium (GE Healthcare, Logan, UT). The stock SHIV.CH505 had a concentration of 178 ng/ml of p27Ag, a titer of 6.8 × 10^6^ IU/ml on TZM cells and 6.32 × 10^8^ vRNA molecules/ml (3.16 × 10^8^ virions/ml). Plasma viral load was determined by real-time reverse transcription-PCR (RT-PCR) using primers specific for SIV/SHIV (Gag region), as previously described^28,69,70^. Weekly SHIV challenges continued until an animal tested positive for SHIV infection by RT-PCR, after which the challenges were stopped, and post-infection sampling proceeded.

### 16s rRNA sequencing and analysis

DNA was extracted from snap-frozen colon biopsies using the PowerFecal DNA Isolation Kit (Qiagen, Valencia, CA) as per the manufacturer’s instructions. Sequencing libraries were created following the Earth Microbiome Protocol for 16 s sequencing^71^. Previously described 515 F (5′-GTGYCAGCMGCCGCGGTAA-3′)—806 R (5′-GGACTACNVGGGTWTCTAAT-3′) primers^72^ were employed to sequence the V3-V4 region of the 16s SSU rRNA. Triplicate amplicon concentrations were normalized, pooled, and cleaned before performing KAPA quantification (KAPA Biosystems, Wilmington, MA). The library was sequenced with an Illumina MiSeq (MiSeq Control Software, including MiSeq Reporter, version 3.1) 2 × 150 bp cycle run utilizing 20% PhiX phage as a control. 16s sequencing reads were demultiplexed then processed using QIIME2 (version 2019.4)^73^. 16s analysis was completed as previously described^74^. Operational taxonomic units (OTUs) were clustered at 99% similarity using the DADA2 method^75^ and assigned taxonomy with the Silva 132 classifier for taxonomic determination^76^. Alpha-diversity (richness, evenness (Pielou)) was calculated using the microbiome package (version 1.8.0), beta-diversity was calculated using pairwise sample dissimilarity and weighted unifrac ordination analysis was performed using principal coordinates analysis (PCoA) using the vegan package for ordination, diversity, and dissimilarities (version 2.5-6), all in RStudio (version 1.2.5033). Statistical analyses were completed using the Pairwise Adonis package (version 0.0.1), and the FSA package (version 0.8.30). Differential abundance analysis was completed using DESeq2^77^ (version 1.26.0). Alpha-diversity, beta-diversity, and taxonomic plots were created in part with RStudio utilizing the phyloseq (version 1.30.0) and ggplot (version 3.2.1) packages.

### Flow cytometry

Multicolor flow cytometric analysis was performed on cellular suspensions of the colon, rectum, and LN tissue and PBMCs according to standard procedures using optimized anti-macaque or anti-human monoclonal antibodies that cross-react with rhesus macaques, as previously described^28^. All samples were first stained with a Live/Dead Fixable Aqua dead cell stain kit (Thermo Fisher Scientific, Grand Island, NY) in order to exclude dead cells. Surface staining of samples was performed using predetermined optimal concentrations of the following antibody-fluorochrome conjugates, with clones listed in parentheses: CD45-PE (phycoerythrin)-CF594 or -BV786 (D058-1283; BD Biosciences, San Jose, CA); CD3-PerCP (peridinin chlorophyll protein) or -BV650 (SP34-2; BD Biosciences); CD4-BV605 (OKT4; BioLegend, San Diego, CA); CD8-APC-H7 (SK1; BD Biosciences) or -BV786 (RPA-T8; BioLegend); CD20-BV570 (2H7; BioLegend); HLA-DR-BV711 (L243; BioLegend); CCR5-PE (3A9; BD Biosciences); CCR6-BV650 (11A9; BD Biosciences); CD28-ECD (CD28.2; Beckman Coulter, Brea, CA); CD95-eFluor450 (DX2; eBioscience/Thermo Fisher Scientific, Waltham, MA); CCR7-FITC (fluorescein isothiocyanate; 3D12; BD Biosciences); IgA-APC (polyclonal; Jackson Immunoresearch, West Grove, PA); and IgG-PE-Cy5 (G18-145; BD Biosciences). The CytoFix/Perm kit (BD Pharmingen) was used to permeabilize and fix cells, after which samples were intracellularly stained with anti-Ki-67-AF700 (BD56; BD). Intracellular cytokine production was assessed by overnight stimulation of colon cells with PMA (5 ng/ml; Sigma-Aldrich) and ionomycin (1 µM/ml; Thermo Fisher Scientific) for 10–14 h, in the presence of brefeldin A (1 µg/ml; Sigma-Aldrich). After overnight incubation, cells were collected, surface stained, permeabilized, and intracellularly stained with the following antibodies: TNF-α-AF700 (Mab11; eBioscience); IL-17A-PE (ebio64CAP17; eBioscience); IL-22-PerCP-eFluor710 (IL22JOP; eBioscience); IFN-γ-BV650 (4S.B3; BioLegend); IL-10-PE-Cy7 (JES3-9D7; BioLegend); IL-21-BV421 (3A3-N2.1; BD Biosciences).

SIV/HIV peptide-specific T cell responses were evaluated by stimulating PBMCs or jejunum cells with 15-mer peptides with 11-amino acid overlap and assessing intracellular cytokine production, as previously described^28^. Briefly, cells were stimulated with SIVmac239 Gag (6204) and HIV-1 consensus C Env (9499), both from the NIH AIDS reagent program, in the presence of CD107a-PE-Cy5 for 1–2 h. Brefeldin A was then added, and cultures incubated overnight. Cells cultured with DMSO or PMA/ionomycin were used as negative and positive controls, respectively. After overnight culture, the True-Nuclear transcription factor buffer set (BioLegend) was used to permeabilize and fix cells, and PBMCs and jejunum cells were stained with the following antibodies: TNF-α-PE-Cy7 (Mab11; BD Biosciences); IFN-γ-FITC (B27; BD Biosciences); IL-2-AF700 (MQ1-17H12; BioLegend). PBMCs were further assessed for expression of CD107a and Granzyme B using the following antibodies: CD107a-PE-Cy5 (eBioH4A3; eBioscience); Granzyme B-BV421 (GB11; BD Biosciences). Peptide-specific cytokine responses are reported after subtraction of DMSO-negative control baseline values. Boolean gating was used to determine the total frequency of cytokine-positive PBMCs in response to peptide stimulation.

All samples were resuspended in 1% paraformaldehyde and held at 4 °C until acquisition. The cytometric acquisition was performed on a BD LSRII cytometer using FACS Diva software (version 8; BD Pharmingen). Analysis of the acquired data was performed using FlowJo software (version 9.9.6 or 10.0.8; Tree Star, Ashland, OR). Individual cell subsets with less than 100 events in the parent gate were not included in analyses due to an inability to ensure adequate fluorescence separation and therefore accurate gating of the population.

### ELISA antibody assays

HIV-1 Env-binding antibody titers were measured in plasma and rectal weck-cel samples collected at specified time points against autologous CAP257 54 wpi_D gp140, as previously described^28,67,78^.

### Neutralization assay

Neutralizing antibodies were assessed using the TZM-bl neutralization assay and HIV-SF162, HIV-JRCSF, HIV-MW965, HIV-CAP257 54wpi_D, and SHIV.CH505 viruses, as previously described^28,63^. All values were calculated with respect to virus-only wells ((relative light unit (RLU) value for virus only − cells only) − (value for serum − cells only))/(value for the virus − cells only).

### Data and statistical analysis

Statistical analyses of 16s microbiome data were performed using RStudio. For alpha-diversity, statistical significance within each experimental group over time and between groups at each time point was assessed using the Dunn’s Kruskal–Wallis multiple comparisons test, adjusted with the Holm method. For beta-diversity, statistical significance within each experimental group over time and between groups at each time point was assessed using the pairwise permutational multivariate analysis of variance (PERMANOVA; Adonis) test. For taxonomic relative abundance, we utilized DESeq2 to determine significant differences in relative abundance of bacterial taxa within each experimental group over time and between groups at each time point. The DESeq2 package is sensitive to sequencing depth and accounts for multiple comparisons using the Benjamini and Hochberg method to control the false discovery rate (FDR) and adjusted *P* values of <0.05 were considered significant. HIV Envelope published sequences were compared using Genbank tools (hiv.lanl.gov). All other statistical analyses were performed using GraphPad Prism statistical software (Version 8; GraphPad Software, San Diego, CA). For SIV and HIV peptide-specific cytokine responses, statistical differences in the total frequency of cytokine-producing or cytolytic effector CD4^+^ and CD8^+^ cells between Pre-Vax (week −2) and week 28 were calculated using a two-tailed Wilcoxon matched-pair signed-rank test. Statistical differences between CD4^+^ and CD8^+^ T cells with individual cytokine or cytolytic effector functions between Pre-Vax and week 28 were calculated using a multiple *t* test with corrections for multiple comparisons using the Holm-Sidak method. Significant differences in survival curves during rectal SHIV.CH505 challenge was calculated using the log-rank (Mantel–Cox) test. Simple linear regressions were performed between the number of rectal SHIV challenges to infection and immune parameters or the relative abundance of colonic bacterial phyla and genera. For all other data, statistical significance between experimental groups at each time point, or within each group between the Pre-PBio baseline (average of weeks −7 and −5), Pre-Vax (week −2) or Pre-SHIV time point (week 28 for experimental groups and the average of weeks −9, −7, and −4 for the control group) and each subsequent time point was assessed using a two-way repeated-measures mixed-effects model with the Geisser–Greenhouse correction and a Tukey’s multiple comparisons tests, with individual variances computed for each comparison. All reported *P* values were multiplicity adjusted and values of <0.05 was considered significant.

### Reporting summary

Further information on research design is available in the Nature Research Reporting Summary linked to this article.

## Supplementary information

Supplementary Data

Reporting Summary

## Data Availability

16s rRNA gene sequence data are available through the NCBI Sequence Read Archive (accession number PRJNA690121). All other data that support the findings of this study are available from the corresponding authors upon reasonable request.
