# Supplementary material for: Effects of persistent modulation of intestinal microbiota on SIV/HIV vaccination in rhesus macaques
Source: NPJ Vaccines. 2021 Mar 11;6:34. doi: 10.1038/s41541-021-00298-4 (PMC7952719; doi:10.1038/s41541-021-00298-4)
Supplement: Supplementary file 1 — Supplementary Data [file 41541_2021_298_MOESM1_ESM.pdf]

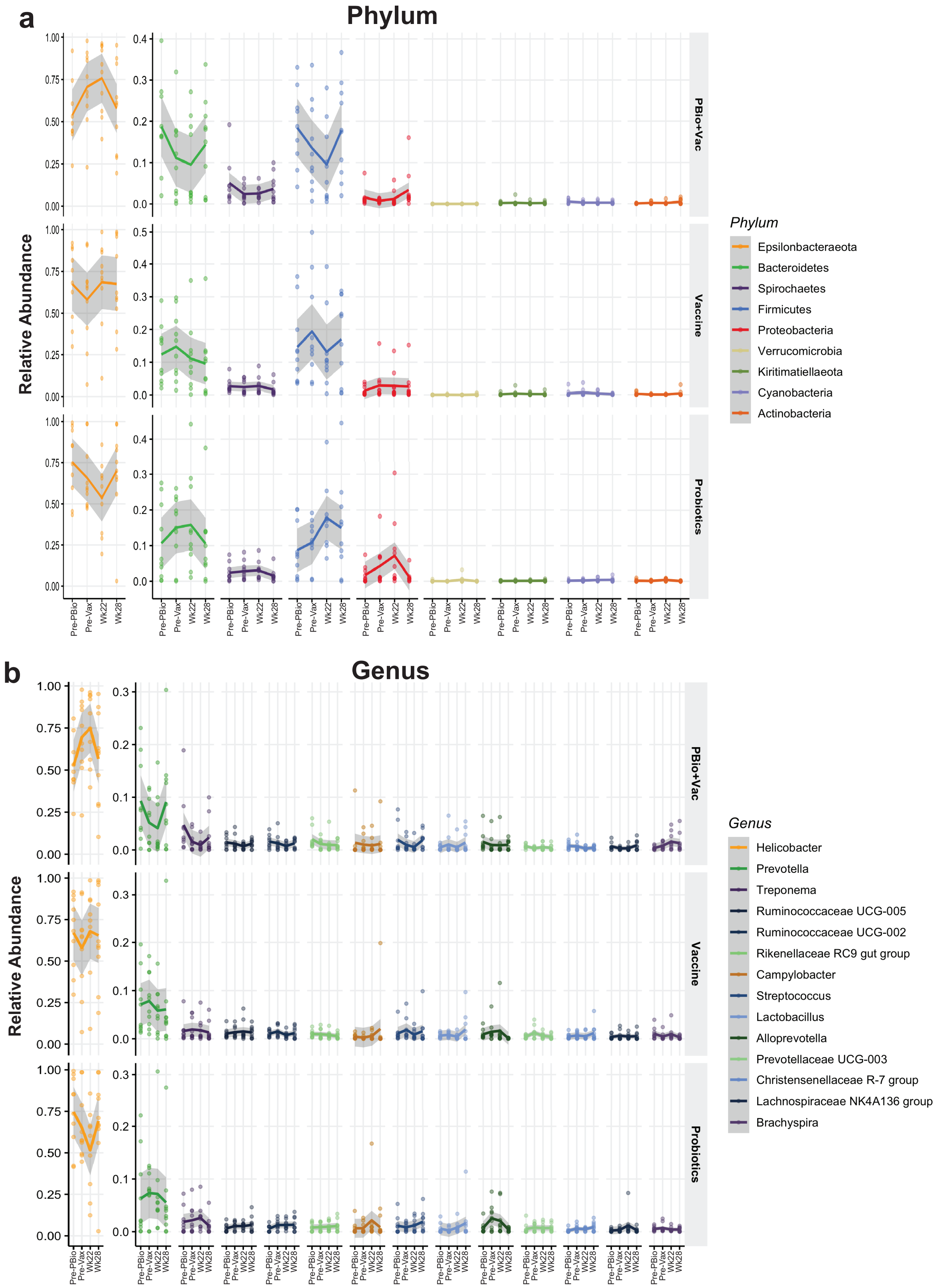

Supplementary Figure 1. Microbial phyla and genera remain consistent in colonic tissue during probiotic administration, SIV/HIV vaccination or combination Probiotics+Vaccine. 16s rRNA gene sequencing was used to characterize microbial communities in Probiotics+Vaccine (n=10), Vaccine only (n=10) and Probiotics only animals (n=10). (a-b) Smoothed mean relative abundance of bacterial phyla (a) and genera (b) at each of the indicated time points. Each phyla or genera are separated out into individual columns. Given that Epsilonbacteraeota and Helicobacter were the dominant communities at the phylum and genus levels, these communities were shown on a different relative abundance scale, in an effort to aid in visualizing the trends in each of the other communities. Solid colored lines represent the mean abundance for specific bacterial phyla. Grey shading overlaying each colored line represents standard error bounds. Matched colored dots surrounding each colored line represent the specific abundances of each bacterial phyla or genera for individual animals. Pre-PBio baseline is an average of week -7 and -5, while Pre-Vax is week -2.

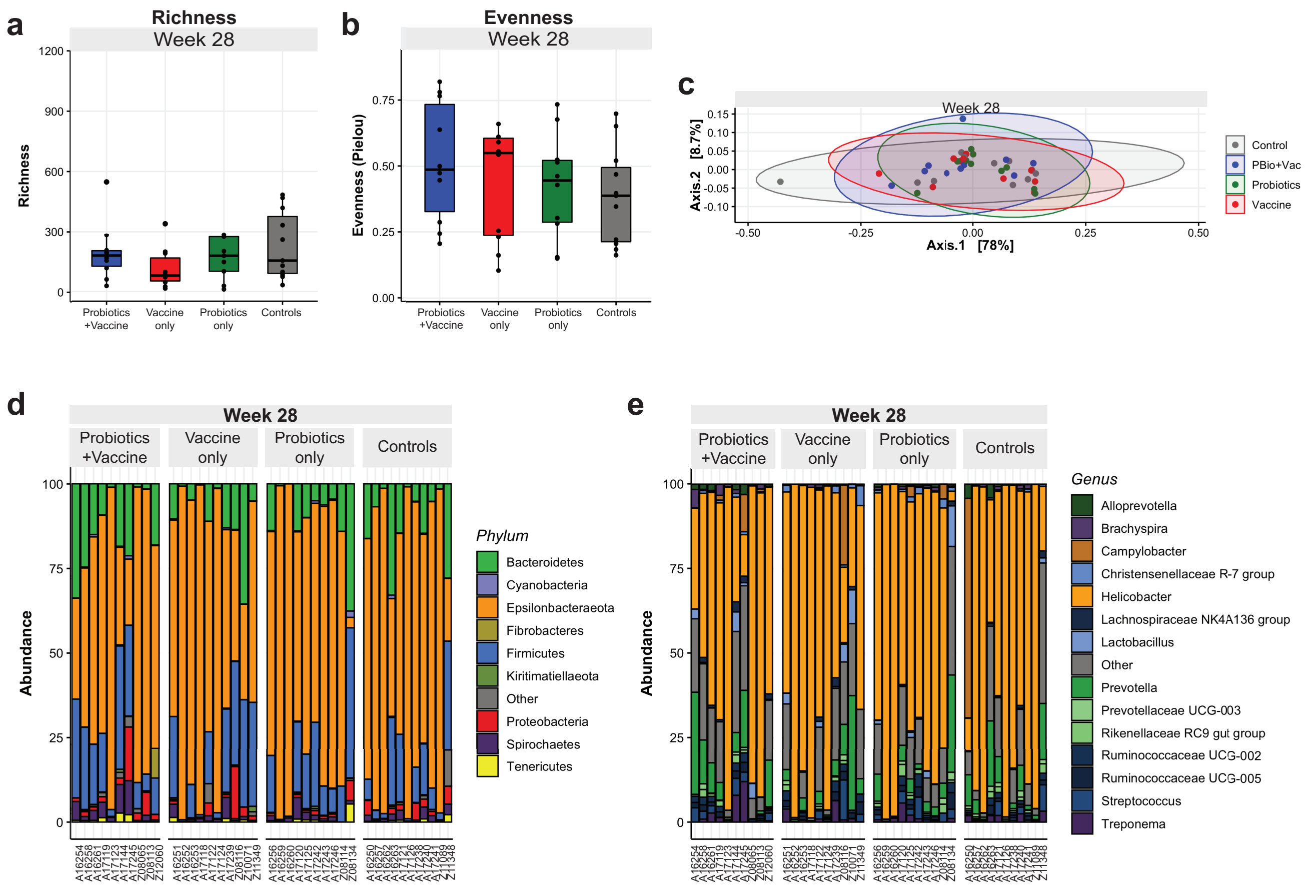

Supplementary Figure 2. Microbial communities are similar in all groups at the last time point prior to SHIV challenge. 16s rRNA gene sequencing was used to characterize microbial communities in Probiotics+Vaccine (n=10), Vaccine only (n=10), Probiotics only animals (n=10) and no Probiotics/no Vaccine control animals at week 28. (a-b) Bacterial community richness (a) and evenness (b) at week 28. Box and whisker bars represent 25-75 percentile and minimum and maximum number of observed OTUs. Black dots that overlay box and whisker plots represent the total number of observed OUTs for individual animals at each time point. Horizontal bars within each box represent the median. (c) Principal components analysis of beta diversity at week 28. Probiotics+Vaccine animals are shown in blue, Vaccine only in red, Probiotics only in green, and no Probiotics/no Vaccine controls in grey. Shaded ovals for each group represent data ellipses. (d-e) Relative abundance taxonomic plots of microbial phyla (d) and genera (e) at week 28. Vertical colored bars represent the percentage of total sequences for specific phyla in individual animals at each of the indicated time points.

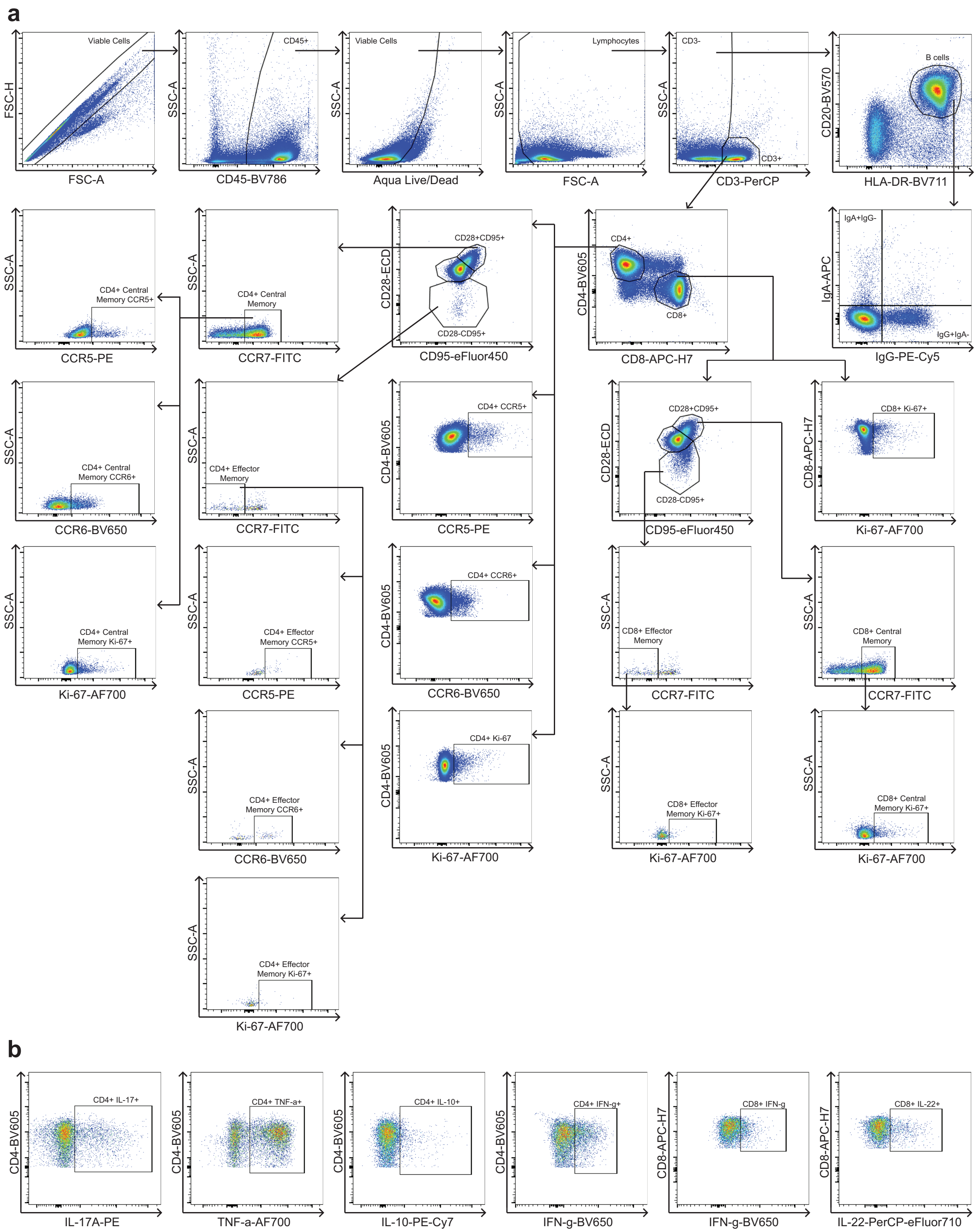

Supplementary Figure 3. Gating strategy used to identify CD4+, CD8+ and B cell subsets. (a) Representative flow plots of stained lymph node cells from a SHIV-uninfected rhesus macaque. Cells were identified by first excluding doublets using forward scatter (FSC) area and height properties, gating on CD45+ cells, excluding dead cells using an Aqua Live/Dead viability dye, and removing any remaining debris using FSC and side scatter (SSC) properties. CD3- cells were identified and B cells were gated as CD20+HLA-DR+ cells. IgA and IgG expressing B cells were identified within CD20+HLA-DR+ B cells. CD3+ cells were identified and CD4+ and CD8+ T cells were gated within CD3+ cells. Total CD4+ cells were assessed for expression of CCR5, CCR6 and Ki-67. Within CD4+ T cells, central memory cells were identified by gating on CD28+CD95+ cells and further classified as CCR7+. Effector memory cells were identified as CD28-CD95+ and further classified as CCR7-. Central and effector memory CD4+ T cells were each assessed for expression of CCR5, CCR6 and Ki-67. Total CD8+ cells were assessed for expression of Ki-67. Central memory cells (CD28+CD95+CCR7+) and effector memory cells (CD28-CD95+CCR7-) were identified within CD8+ T cells. Central and effector memory CD8+ T cells were assessed for expression of Ki-67. (b) CD4+ and CD8+ T cells producing cytokines were characterized subsequent to mitogenic PMA-ionomycin stimulation in colon tissue. Representative flow plots demonstrate gating to identify the percentage of IL-17, TNF-a IL-10 and IFN-g producing CD4+ T cells and IFN-g and IL-22 producing CD8+ T cells.

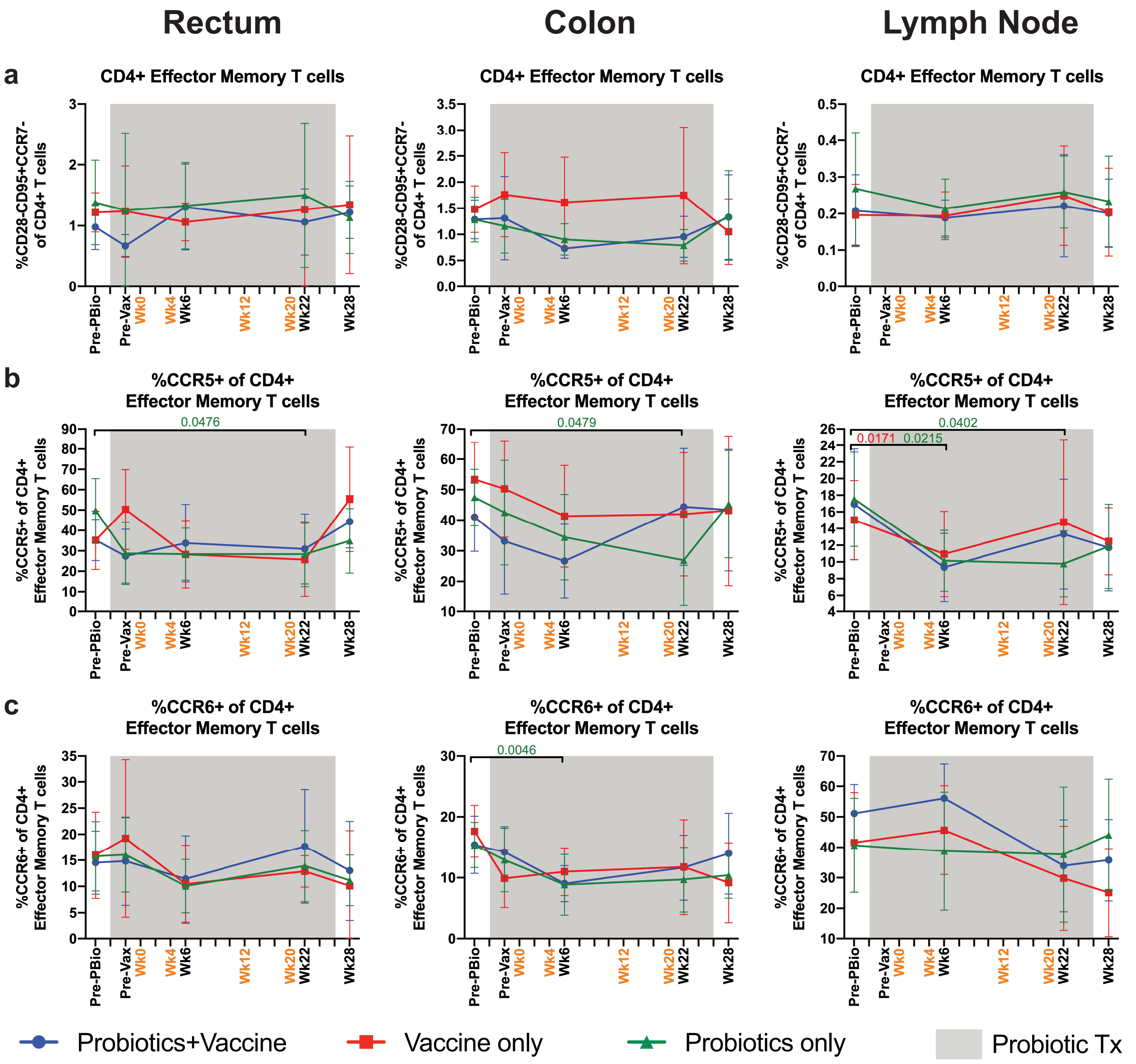

Supplementary Figure 4. Frequency of effector memory T cells, including CCR5+ and CCR6+ expressing cells, in mucosal and lymph node tissue during probiotic administration, SIV/HIV vaccination or combination Probiotics+Vaccine. Effector memory CD4+ T cell subsets expressing CCR5 or CCR6 were characterized in the rectum, colon and lymph node of Probiotics+Vaccine (n=10), Vaccine only (n=10) and Probiotics only (n=10) treated animals by flow cytometry. (a) Percentage of CD4+ effector memory cells (CD28-CD95+CCR7-) of CD4+ T cells. (b) Percentage of CCR5+ cells of CD4+ effector memory T cells. (c) Percentage of CCR6+ cells of CD4+ effector memory T cells. In all panels, data is depicted as the mean and 95% confidence interval for each group: Probiotics+Vaccine=blue circles, Vaccine only=red squares, Probiotics only=green triangles. Pre-PBio baseline is an average of week -7 and -5, while Pre-Vax is week -2. Immunizations at weeks 0, 4, 12 and 20 are indicated in orange font. Daily oral probiotics were administered between week -5 and week 26, indicated by the grey bar. For comparisons within each group between Pre-PBio and subsequent time points, multiplicity adjusted significant p values are shown above horizontal black bars, with fonts colored to indicate the experimental group.

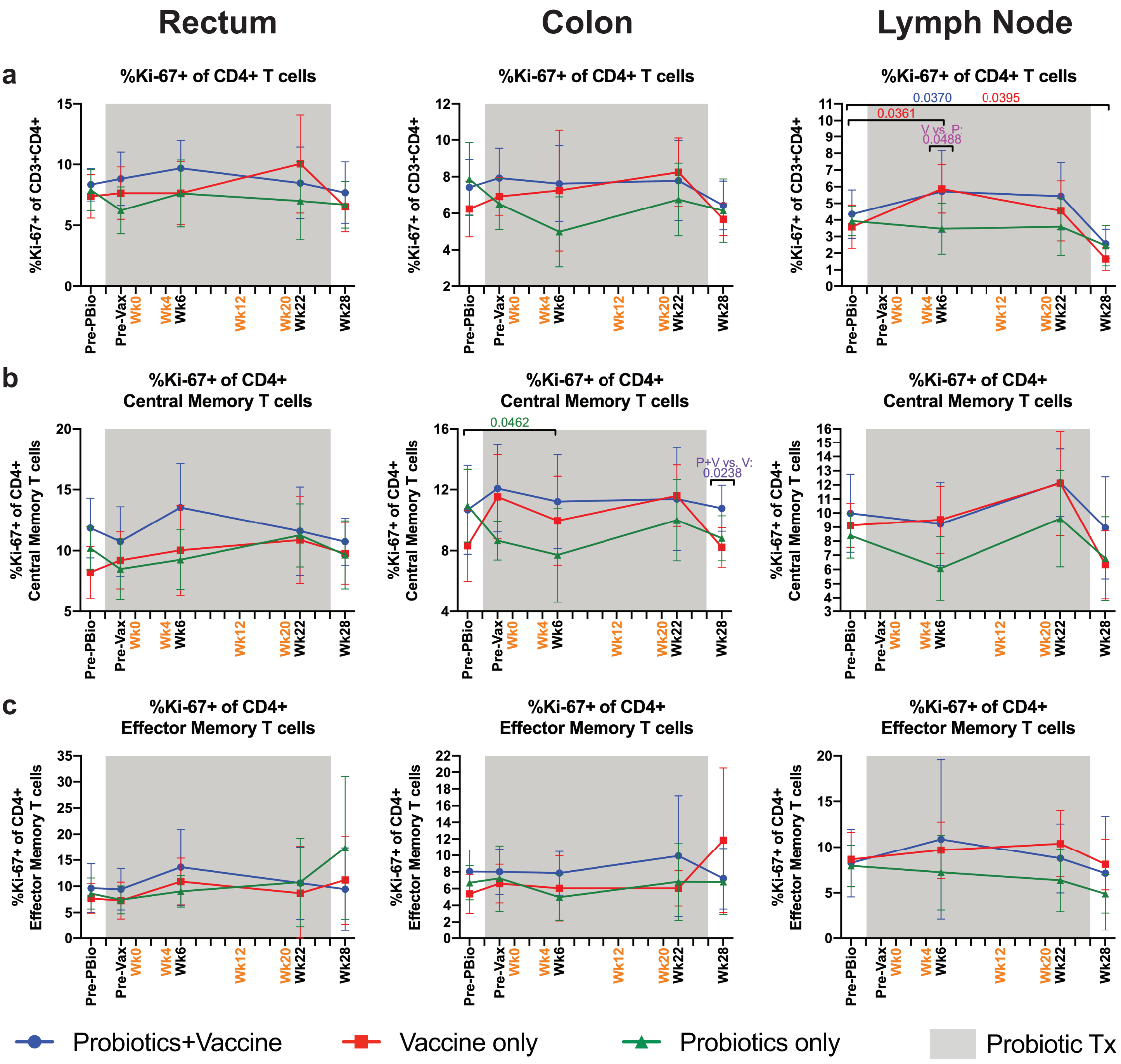

Supplementary Figure 5. Frequency of Ki-67+ CD4+ T cell subsets in mucosal and lymph node tissue during probiotic administration, SIV/HIV vaccination or combination Probiotics+Vaccine. CD4+ T cell subsets expressing Ki-67 were characterized in the rectum, colon and lymph node of Probiotics+Vaccine (n=10), Vaccine only (n=10) and Probiotics only (n=10) treated animals by flow cytometry. (a) Percentage of Ki-67+ cells of CD4+ T cells. (b) Percentage of Ki-67+ cells of CD4+ central memory T cells. (c) Percentage of Ki-67+ cells of CD4+ effector memory T cells. In all panels, data is depicted as the mean and 95% confidence interval for each group: Probiotics+Vaccine=blue circles, Vaccine only=red squares, Probiotics only=green triangles. Pre-PBio baseline is an average of week -7 and -5, while Pre-Vax is week -2. Immunizations at weeks 0, 4, 12 and 20 are indicated in orange font. Daily oral probiotics were administered between week -5 and week 26, indicated by the grey bar. For comparisons within each group between Pre-PBio and subsequent time points, multiplicity adjusted significant p values are shown above horizontal black bars, with fonts colored to indicate the experimental group. For comparisons between groups at each time point, multiplicity adjusted significant p values are specified above the designated time point.

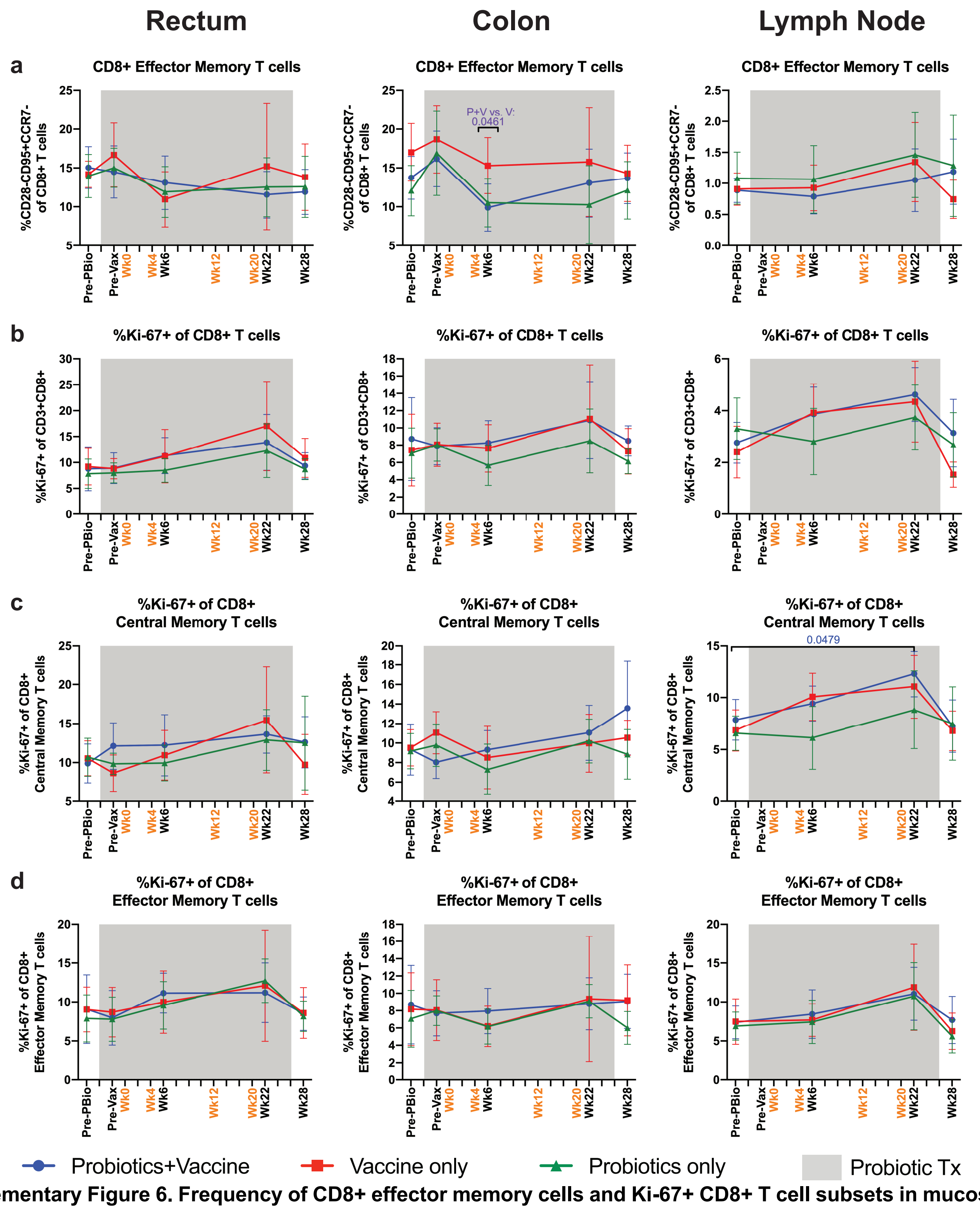

Supplementary Figure 6. Frequency of CD8+ effector memory cells and Ki-67+ CD8+ T cell subsets in mucosal and lymph node tissue during probiotic administration, SIV/HIV vaccination or combination Probiotics+Vaccine. CD8+ T cell subsets expressing Ki-67 were characterized in the rectum, colon and lymph node of Probiotics+Vaccine (n=10), Vaccine only (n=10) and Probiotics only (n=10) treated animals by flow cytometry. (a) Percentage of CD8+ effector memory cells (CD28-CD95+CCR7-) of CD8+ T cells. (b) Percentage of Ki-67+ cells of CD8+ T cells. (c) Percentage of Ki-67+ cells of CD8+ central memory T cells. (d) Percentage of Ki-67+ cells of CD8+ effector memory T cells. In all panels, data is depicted as the mean and 95% confidence interval for each group: Probiotics+Vaccine=blue circles, Vaccine only=red squares, Probiotics only=green triangles. Pre-PBio baseline is an average of week -7 and -5, while Pre-Vax is week -2. Immunizations at weeks 0, 4, 12 and 20 are indicated in orange font. Daily oral probiotics were administered between week -5 and week 26, indicated by the grey bar. For comparisons within each group between Pre-PBio and subsequent time points, multiplicity adjusted significant p values are shown above horizontal black bars, with fonts colored to indicate the experimental group. For comparisons between groups at each time point, multiplicity adjusted significant p values are specified above the designated time point.

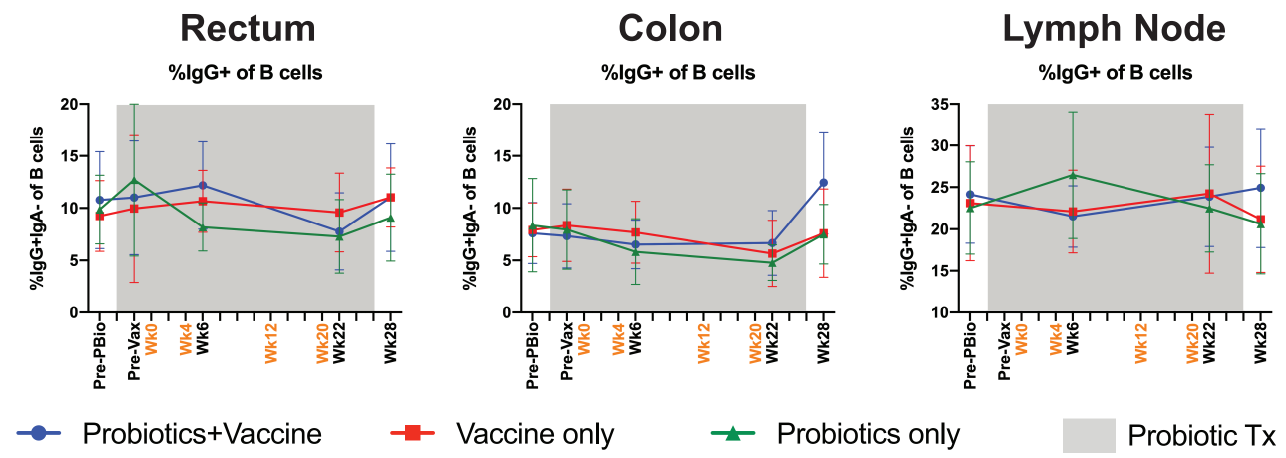

Supplementary Figure 7. IgG+ B cell frequencies in mucosal and lymph node tissue during probiotic administration, SIV/HIV vaccination or combination Probiotics+Vaccine. IgG+ B cell frequencies were characterized in the rectum, colon and lymph node of Probiotics+Vaccine (n=10), Vaccine only (n=10) and Probiotics only (n=10) treated animals by flow cytometry. In all panels, data is depicted as the mean and 95% confidence interval for each group: Probiotics+Vaccine=blue circles, Vaccine only=red squares, Probiotics only=green triangles. Pre-PBio baseline is an average of week -7 and -5, while Pre-Vax is week -2. Immunizations at weeks 0, 4, 12 and 20 are indicated in orange font. Daily oral probiotics were administered between week -5 and week 26, indicated by the grey bar.

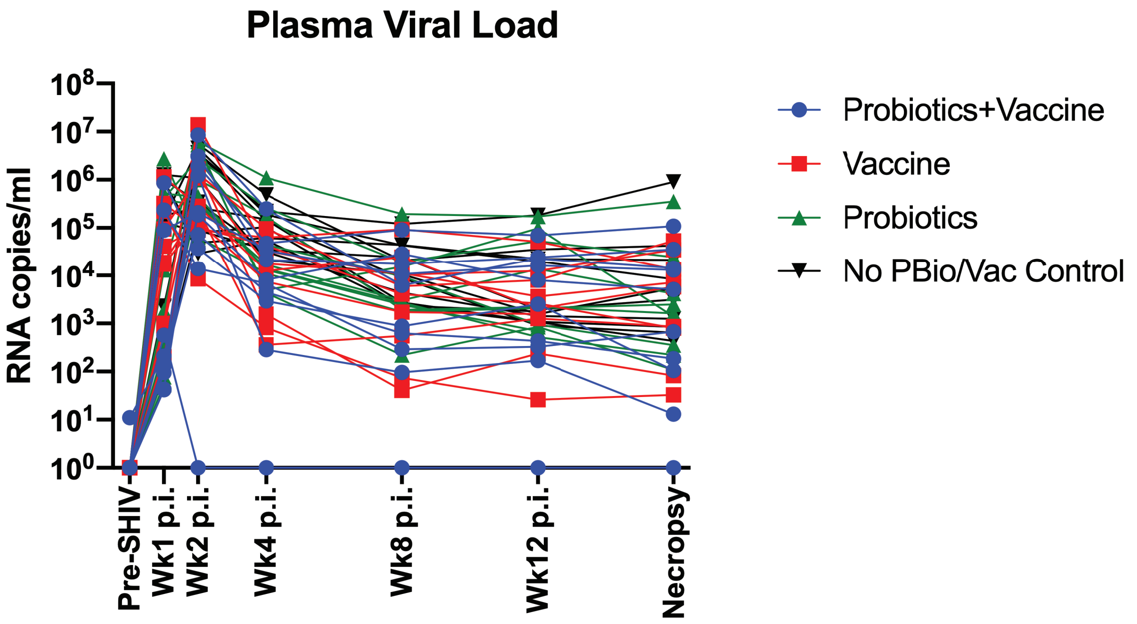

Supplementary Figure 8. Plasma viral load after SHIV.CH505 infection. Post-infection (p.i.) viral load in plasma was measured after SHIV.CH505 infection of Probiotics+Vaccine (n=10), Vaccine only (n=10), Probiotics only (n=10) and no Probiotics/no Vaccine controls (n=11). Each animal is indicated by a single point, with lines connecting data from the same animal and colors indicating different groups: Probiotics+Vaccine=blue circles, Vaccine only=red squares, Probiotics only=green triangles. Data at Pre-SHIV time point is Week28 for Probiotics+Vaccine, Vaccine only and Probiotics only, and an average of Week-9, -7 and -4 for no Probiotics/no Vaccine control animals.

Supplementary Table 1. Mean (95% CI[1]) and p values for statistical analysis of differences in CD4+ T cell subset frequencies between Pre-Vax and subsequent time points in rectum and colon tissue

			Probiotics+Vaccine		Vaccine only		Probiotics only	
			Mean (95% CI[1])	p value[2]	Mean (95% CI[1])	p value[2]	Mean (95% CI[1])	p value[2]
Rectum	CD4+ T cells	Pre-PBio	15.35 (11.61, 19.08)		14.56 (10.85, 18.27)		13.63 (10.4, 16.86)	
		Pre-Vax	17.04 (12.28, 21.79)		15.57 (12.81, 18.33)		16.61 (12.51, 20.71)	
		Wk6	13.65 (10.76, 16.54)	0.1647	18.33 (14.5, 22.17)	0.3057	14.83 (12.38, 17.28)	0.7045
		Wk22	12.29 (9.88, 14.69)	0.1837	11.57 (5.43, 17.72)	0.4586	14.07 (10.26, 17.88)	0.8077
		Wk28	12.58 (9.94, 15.22)	0.4322	11.11 (8.66, 13.56)	0.1073	13.29 (9.15, 17.44)	0.4969
	CD4+ Central Memory T cells	Pre-PBio	35.83 (28.87, 42.78)		40.09 (44.54, 35.63)		39.29 (33.79, 44.78)	
		Pre-Vax	33.62 (27.63, 39.61)		39.62 (34.88, 44.36)		40.56 (32.84, 48.28)	
		Wk6	37.22 (31.91, 42.53)	0.7704	42.88 (36.24, 49.52)	0.8521	38.54 (34.64, 42.44)	0.9861
		Wk22	33.35 (23.50, 43.20)	>0.9999	37.79 (30.73, 44.85)	0.9774	32.34 (24.80, 39.89)	0.4133
		Wk28	39.13 (31.91, 46.35)	0.1343	44.80 (39.03, 50.57)	0.5404	37.11 (32.54, 41.68)	0.8923
	CD4+ Effector Memory T cells	Pre-PBio	0.98 (0.61, 1.35)		1.22 (0.90, 1.54)		1.38 (0.69, 2.07)	
		Pre-Vax	0.67 (0.48, 0.85)		1.24 (1.98, 0.50)		1.25 (-0.01, 2.52)	
		Wk6	1.31 (0.60, 2.01)	0.4039	1.06 (0.75, 1.37)	0.9017	1.33 (0.62, 2.04)	0.9994
		Wk22	1.06 (0.52, 1.60)	0.5084	1.27 (-0.15, 2.69)	>0.9999	1.50 (0.32, 2.69)	0.9424
		Wk28	1.22 (0.79, 1.65)	0.3271	1.34 (0.22, 2.47)	0.9997	1.14 (0.54, 1.73)	0.9992
Colon	CD4+ T cells	Pre-PBio	15.24 (12.51, 17.98)		15.99 (13.55, 18.43)		16.10 (13.30, 18.90)	
		Pre-Vax	16.60 (13.31, 19.89)		13.22 (10.00, 16.43)		15.27 (11.54, 18.99)	
		Wk6	19.03 (14.56, 23.50)	0.7030	15.34 (11.63, 19.05)	0.6294	20.07 (16.92, 23.22)	0.1131
		Wk22	15.73 (12.92, 18.54)	0.9889	12.97 (10.43, 15.51)	0.9997	18.94 (16.14, 21.74)	0.5812
		Wk28	14.46 (9.86, 19.07)	0.7458	14.85 (10.06, 19.64)	0.9710	14.95 (11.61, 18.29)	>0.9999
	CD4+ Central Memory T cells	Pre-PBio	35.17 (30.09, 40.25)		33.07 (28.30, 37.83)		38.43 (33.21, 43.64)	
		Pre-Vax	40.22 (34.89, 45.55)		34.06 (24.91, 43.21)		40.70 (34.36, 47.04)	
		Wk6	42.14 (37.68, 46.60)	0.9686	40.86 (33.40, 48.32)	0.4757	39.85 (36.27, 43.43)	0.9987
		Wk22	38.53 (26.91, 50.16)	0.9981	36.69 (30.05, 43.32)	0.9475	35.98 (31.85, 40.11)	0.6589
		Wk28	40.69 (35.55, 45.83)	>0.9999	43.74 (38.03, 49.45)	0.4380	38.25 (34.97, 41.53)	0.7734
	CD4+ Effector Memory T cells	Pre-PBio	1.29 (0.92, 1.66)		1.48 (1.04, 1.93)		1.28 (0.85, 1.71)	
		Pre-Vax	1.31 (0.52, 2.11)		1.76 (0.96, 2.56)		1.16 (0.64, 1.68)	
		Wk6	0.73 (0.54, 0.92)	0.4850	1.61 (0.75, 2.48)	0.9961	0.90 (0.60, 1.20)	0.7867
		Wk22	0.95 (0.56, 1.35)	0.9360	1.75 (0.44, 3.06)	>0.9999	0.79 (0.49, 1.09)	0.5255
		Wk28	1.33 (0.52, 2.14)	>0.9999	1.05 (0.43, 1.68)	0.6180	1.36 (0.51, 2.22)	0.9757

[1]CI: Confidence Interval.

[2]Multiplicity adjusted p values indicating the statistical significance between the Pre-Vax time point and subsequent time points. Statistical significance calculated using a two-way repeated measures mixed-effects model with the Geisser-Greenhouse correction and a Tukey's multiple comparisons test, with individual variances computed for each comparison.

Supplementary Table 2. Mean (95% CI[1]) and p values for statistical analysis of differences in %CCR5+ of CD4+ T cell subset frequencies between Pre-Vax and subsequent time points in rectum and colon tissue

			Probiotics+Vaccine		Vaccine only		Probiotics only	
			Mean (95% CI[1])	p value[2]	Mean (95% CI[1])	p value[2]	Mean (95% CI[1])	p value[2]
Rectum	%CCR5+ of CD4+ T cells	Pre-PBio	34.76 (29.52, 40.00)		29.63 (22.08, 37.17)		42.73 (29.80, 55.65)	
		Pre-Vax	26.50 (17.25, 35.75)		34.26 (20.69, 47.83)		33.23 (18.03, 48.42)	
		Wk6	33.86 (22.17, 45.55)	0.6819	24.72 (15.95, 33.49)	0.2682	24.30 (14.04, 34.56)	0.5827
		Wk22	22.12 (14.27, 29.97)	0.7863	28.41 (12.21, 44.62)	0.9349	23.11 (11.88, 34.34)	0.2750
		Wk28	37.70 (26.21, 49.19)	0.3048	41.86 (25.94, 57.78)	0.7373	30.54 (16.40, 44.69)	0.7522
	%CCR5+ of CD4+ Central Memory T cells	Pre-PBio	42.60 (36.80, 48.39)		33.88 (26.67, 41.09)		46.81 (35.31, 58.31)	
		Pre-Vax	32.68 (25.25, 40.11)		37.31 (25.89, 48.73)		36.11 (21.77, 50.45)	
		Wk6	38.89 (28.65, 49.13)	0.7140	29.75 (19.98, 39.52)	0.3024	29.07 (19.36, 38.78)	0.7767
		Wk22	28.51 (16.86, 40.16)	0.9110	33.86 (20.64, 47.09)	0.9687	29.17 (18.55, 39.78)	0.5652
		Wk28	43.95 (31.59, 56.31)	0.3345	44.49 (28.75, 60.23)	0.7623	36.22 (20.97, 51.47)	>0.9999
	% CCR5+ of CD4+ Effector Memory T cells	Pre-PBio	35.29 (25.27, 45.31)		35.19 (20.90, 49.47)		49.73 (34.04, 65.42)	
		Pre-Vax	27.43 (14.22, 40.63)		50.29 (30.72, 69.85)		28.80 (13.54, 44.05)	
		Wk6	33.78 (14.78, 52.77)	0.8842	28.27 (11.78, 44.75)	**0.0361**	28.41 (15.67, 42.15)	>0.9999
		Wk22	30.93 (13.83, 48.04)	0.7180	25.65 (7.78, 43.52)	0.4806	28.37 (12.50, 44.25)	0.9998
		Wk28	44.28 (31.45, 47.10)	0.2410	55.26 (29.63, 80.89)	0.9817	34.88 (19.09, 50.67)	0.5829
Colon	%CCR5+ of CD4+ T cells	Pre-PBio	46.92 (36.45, 57.38)		52.18 (44.88, 59.47)		46.21 (35.63, 56.79)	
		Pre-Vax	35.12 (27.30, 42.94)		46.54 (31.34, 61.74)		44.13 (27.58, 60.68)	
		Wk6	31.63 (22.08, 41.18)	0.9536	46.87 (37.89, 55.85)	>0.9999	32.41 (17.82, 47.00)	0.1781
		Wk22	34.88 (22.13, 47.63)	>0.9999	44.84 (34.49, 55.18)	0.9992	25.36 (13.58, 37.13)	0.1211
		Wk28	46.48 (32.70, 60.25)	0.3469	43.96 (27.17, 60.75)	0.9989	41.27 (27.09, 55.45)	0.9948
	% CCR5+ of CD4+ Central Memory T cells	Pre-PBio	50.17 (40.55, 59.79)		51.85 (43.55, 60.15)		49.38 (39.04, 59.72)	
		Pre-Vax	38.25 (29.61, 46.89)		46.59 (33.69, 59.49)		44.72 (28.84, 60.60)	
		Wk6	36.85 (28.90, 44.80)	0.9962	49.73 (42.18, 57.28)	0.9870	36.39 (23.08, 49.70)	0.2740
		Wk22	38.28 (29.45, 47.11)	>0.9999	45.85 (32.68, 59.02)	>0.9999	32.48 (21.22, 43.74)	0.3271
		Wk28	48.76 (35.31, 62.20)	0.4326	48.72 (33.19, 64.25)	0.9987	45.10 (31.28, 58.92)	>0.9999
	% CCR5+ of CD4+ Effector Memory T cells	Pre-PBio	41.03 (29.89, 52.17)		53.40 (41.14, 65.65)		47.46 (38.22, 56.70)	
		Pre-Vax	33.18 (15.92, 50.43)		50.29 (34.45, 66.13)		42.53 (25.41, 59.65)	
		Wk6	26.68 (14.61, 38.75)	0.7969	41.34 (24.71, 57.97)	0.7954	34.48 (20.53, 48.43)	0.3451
		Wk22	44.39 (25.25, 63.52)	0.5214	41.98 (21.82, 62.13)	0.9329	26.92 (11.96, 41.88)	0.2324
		Wk28	43.35 (23.50, 63.20)	0.4522	43.17 (18.65, 67.68)	0.9856	45.28 (27.75, 62.80)	0.9959

[1]CI: Confidence Interval.

[2]Multiplicity adjusted p values indicating the statistical significance between the Pre-Vax time point and subsequent time points. Statistical significance calculated using a two-way repeated measures mixed-effects model with the Geisser-Greenhouse correction and a Tukey's multiple comparisons test, with individual variances computed for each comparison.

Supplementary Table 3. Mean (95% CI[1]) and p values for statistical analysis of differences in %CCR6+ of CD4+ T cell subset frequencies between Pre-Vax and subsequent time points in rectum and colon tissue

			Probiotics+Vaccine		Vaccine only		Probiotics only	
			Mean (95% CI[1])	p value[2]	Mean (95% CI[1])	p value[2]	Mean (95% CI[1])	p value[2]
Rectum	%CCR6+ of CD4+ T cells	Pre-PBio	23.19 (17.01, 29.37)		20.72 (13.98, 27.46)		22.13 (15.78, 28.47)	
		Pre-Vax	17.97 (12.54, 23.40)		24.85 (33.23, 16.47)		19.03 (8.59, 29.48)	
		Wk6	16.08 (11.86, 20.30)	0.9035	15.21 (9.06, 21.36)	**0.0099**	17.67 (9.60, 25.74)	0.9966
		Wk22	16.77 (9.83, 23.71)	0.9937	17.65 (9.70, 25.59)	0.4582	13.04 (6.86, 19.22)	0.6126
		Wk28	19.66 (9.90, 29.42)	0.9923	18.47 (10.03, 26.90)	0.7541	12.79 (8.79, 16.79)	0.6138
	%CCR6+ of CD4+ Central Memory T cells	Pre-PBio	33.26 (25.65, 40.88)		27.65 (19.65, 35.64)		28.87 (20.85, 36.90)	
		Pre-Vax	25.16 (20.21, 30.11)		31.14 (21.16, 41.12)		22.11 (8.33, 35.88)	
		Wk6	24.67 (17.23, 32.11)	0.9999	19.41 (11.44, 27.39)	0.1555	18.29 (25.63, 10.95)	0.9475
		Wk22	24.03 (16.46, 31.60)	0.9958	25.18 (33.42, 16.94)	0.5866	18.49 (23.77, 13.21)	0.9611
		Wk28	23.89 (14.29, 33.49)	0.9964	23.49 (14.33, 32.65)	0.5805	14.36 (10.63, 18.10)	0.6849
	%CCR6+ of CD4+ Effector Memory T cells	Pre-PBio	14.55 (8.51, 20.60)		15.99 (7.74, 24.23)		15.78 (915, 22.41)	
		Pre-Vax	14.84 (6.40, 23.39)		19.24 (4.15, 34.33)		16.06 (8.94, 23.18)	
		Wk6	11.46 (3.22, 19.69)	0.8613	10.45 (2.99, 17.91)	0.2647	10.08 (5.01, 15.15)	0.2523
		Wk22	17.69 (6.85, 28.53)	0.8618	12.87 (9.88, 15.85)	0.8147	13.92 (7.12, 20.72)	0.9048
		Wk28	13.02 (3.55, 22.48)	0.9862	10.11 (-0.49, 20.71)	0.8487	11.17 (6.34, 15.99)	0.6610
Colon	%CCR6+ of CD4+ T cells	Pre-PBio	22.55 (15.11, 29.98)		30.65 (23.90, 37.39)		24.16 (17.68, 30.65)	
		Pre-Vax	19.58 (13.43, 25.72)		16.75 (10.50, 23.00)		16.65 (9.60, 23.70)	
		Wk6	11.70 (7.22, 16.18)	0.0524	19.79 (13.18, 26.39)	0.8617	15.54 (8.60, 22.47)	0.9829
		Wk22	18.96 (10.35, 27.57)	0.9803	19.65 (11.13, 28.16)	0.6828	14.10 (7.09, 21.12)	0.9075
		Wk28	18.35 (6.05, 30.66)	0.9959	15.75 (5.46, 26.03)	0.9985	12.26 (7.18, 17.35)	0.5748
	%CCR6+ of CD4+ Central Memory T cells	Pre-PBio	29.27 (20.81, 37.72)		38.32 (30.29, 46.34)		32.67 (23.87, 41.46)	
		Pre-Vax	27.35 (17.47, 37.22)		23.77 (13.22, 34.32)		22.51 (14.04, 30.98)	
		Wk6	14.15 (8.26, 20.04)	**0.0209**	24.08 (15.72, 32.43)	>0.9999	22.79 (12.85, 32.72)	>0.9999
		Wk22	25.88 (15.01, 36.76)	0.9322	26.56 (12.57, 40.55)	0.9254	19.82 (11.27, 28.38)	0.9753
		Wk28	25.96 (10.24, 41.68)	0.9968	20.79 (8.08, 33.50)	0.9150	16.20 (10.03, 22.37)	0.2464
	%CCR6+ of CD4+ Effector Memory T cells	Pre-PBio	15.41 (10.70, 20.12)		17.63 (13.37, 21.88)		15.37 (11.66, 19.08)	
		Pre-Vax	14.22 (10.04, 18.40)		9.89 (5.14, 14.64)		12.92 (7.71, 18.13)	
		Wk6	9.03 (6.09, 11.96)	0.0781	10.98 (7.09, 14.88)	0.9856	8.85 (3.90, 13.81)	0.2271
		Wk22	11.67 (6.34, 16.99)	0.7387	11.75 (4.00, 19.51)	0.7358	9.70 (4.42, 14.98)	0.5546
		Wk28	13.95 (7.33, 20.58)	0.9997	9.20 (2.65, 15.74)	0.9983	10.40 (6.66, 14.14)	0.8891

[1]CI: Confidence Interval.

[2]Multiplicity adjusted p values indicating the statistical significance between the Pre-Vax time point and subsequent time points. Statistical significance calculated using a two-way repeated measures mixed-effects model with the Geisser-Greenhouse correction and a Tukey's multiple comparisons test, with individual variances computed for each comparison.

Supplementary Table 4. Mean (95% CI[1]) and p values for statistical analysis of differences in %Ki-67+ of CD4+ T cell subset frequencies between Pre-Vax and subsequent time points in rectum and colon tissue

			Probiotics+Vaccine		Vaccine only		Probiotics only	
			Mean (95% CI[1])	p value[2]	Mean (95% CI[1])	p value[2]	Mean (95% CI[1])	p value[2]
Rectum	%Ki-67+ of CD4+ T cells	Pre-PBio	8.35 (7.02, 9.69)		7.39 (5.60, 9.17)		7.91 (6.23, 9.58)	
		Pre-Vax	8.83 (6.65, 11.01)		7.66 (5.51, 9.80)		6.25 (4.32, 8.17)	
		Wk6	9.69 (7.43, 11.95)	0.9205	7.66 (5.06, 10.25)	>0.9999	7.63 (4.88, 10.37)	0.6650
		Wk22	8.48 (5.55, 11.42)	0.9994	10.06 (6.02, 14.10)	0.5895	7.02 (3.83, 10.21)	0.9930
		Wk28	7.69 (5.16, 10.22)	0.9329	6.55 (4.48, 8.62)	0.9380	6.70 (4.79, 8.62)	0.9947
	%Ki-67+ of CD4+ Central Memory T cells	Pre-PBio	11.85 (9.40, 14.30)		8.23 (6.13, 10.33)		10.20 (8.43, 11.97)	
		Pre-Vax	10.74 (7.88, 13.59)		9.21 (6.88, 11.54)		8.49 (6.03, 10.96)	
		Wk6	13.53 (9.95, 17.11)	0.3085	10.03 (6.35, 13.72)	0.9949	9.26 (6.83, 11.69)	0.9766
		Wk22	11.59 (7.98, 15.21)	0.9873	10.87 (7.33, 14.41)	0.8339	11.25 (8.66, 13.84)	0.5524
		Wk28	10.74 (8.81, 12.66)	>0.9999	9.77 (7.27, 12.26)	0.9612	9.66 (6.88, 12.43)	0.8923
	%Ki-67+ of CD4+ Effector Memory T cells	Pre-PBio	9.64 (5.00, 14.28)		7.70 (4.90, 10.49)		8.62 (5.68, 11.56)	
		Pre-Vax	9.44 (5.49, 13.38)		7.28 (3.77, 10.78)		7.41 (4.78, 10.05)	
		Wk6	13.63 (6.36, 20.91)	0.5606	10.91 (6.46, 15.35)	0.4392	9.03 (6.05, 1.01)	0.9513
		Wk22	10.61 (3.70, 17.51)	0.9902	8.68 (-0.37, 17.73)	0.7977	10.78 (2.33, 19.23)	0.8485
		Wk28	9.44 (1.49, 17.39)	>0.9999	11.19 (2.76, 19.63)	0.7349	17.38 (3.72, 31.03)	0.3772
Colon	%Ki-67+ of CD4+ T cells	Pre-PBio	7.42 (5.91, 8.94)		6.24 (4.71, 7.777)		7.87 (5.88, 9.86)	
		Pre-Vax	7.93 (6.31, 9.55)		6.92 (5.89, 7.95)		6.51 (5.11, 7.91)	
		Wk6	7.62 (5.55, 9.69)	0.9982	7.26 (3.94, 10.57)	0.9995	4.99 (3.08, 6.90)	0.5937
		Wk22	7.79 (5.60, 9.97)	0.9994	8.25 (6.40, 10.10)	0.5931	6.76 (4.77, 8.74)	0.9994
		Wk28	6.43 (5.10, 7.76)	0.3521	5.67 (4.78, 6.57)	0.1343	6.14 (4.41, 7.88)	0.9958
	%Ki-67+ of CD4+ Central Memory T cells	Pre-PBio	10.67 (7.76, 13.59)		8.31 (5.99, 10.63)		10.94 (8.56, 13.33)	
		Pre-Vax	12.08 (9.22, 14.93)		11.53 (8.77, 14.28)		8.65 (7.38, 9.93)	
		Wk6	11.21 (8.13, 14.29)	0.9935	9.96 (7.04, 12.87)	0.6794	7.72 (4.65, 10.78)	0.9711
		Wk22	11.39 (8.02, 14.76)	0.9953	11.60 (9.58, 13.62)	>0.9999	9.99 (7.33, 12.65)	0.8385
		Wk28	10.78 (9.27, 12.29)	0.8208	8.21 (6.92, 9.51)	0.1924	8.81 (7.34, 10.29)	0.9996
	%Ki-67+ of CD4+ Effector Memory T cells	Pre-PBio	8.03 (5.39, 10.68)		5.38 (3.05, 7.70)		6.71 (4.67, 8.75)	
		Pre-Vax	8.02 (5.28, 10.75)		6.61 (4.31, 8.91)		7.23 (3.30, 11.16)	
		Wk6	7.85 (5.22, 10.49)	>0.9999	6.04 (2.16, 9.93)	0.9955	4.97 (2.22, 7.72)	0.8444
		Wk22	9.93 (2.68, 17.17)	0.9270	6.04 (3.93, 8.14)	0.9922	6.83 (2.21, 11.45)	0.9998
		Wk28	7.21 (3.57, 10.84)	0.9956	11.85 (3.14, 20.56)	0.5264	6.81 (2.92, 10.69)	0.9995

[1]CI: Confidence Interval.

[2]Multiplicity adjusted p values indicating the statistical significance between the Pre-Vax time point and subsequent time points. Statistical significance calculated using a two-way repeated measures mixed-effects model with the Geisser-Greenhouse correction and a Tukey's multiple comparisons test, with individual variances computed for each comparison.

Supplementary Table 5. Mean (95% CI[1]) and p values for statistical analysis of differences in CD8+ T cell subset frequencies between Pre-Vax and subsequent time points in rectum and colon tissue

			Probiotics+Vaccine		Vaccine only		Probiotics only	
			Mean (95% CI[1])	p value[2]	Mean (95% CI[1])	p value[2]	Mean (95% CI[1])	p value[2]
Rectum	CD8+ T cells	Pre-PBio	17.99 (13.62, 22.36)		15.99 (11.96, 20.02)		17.55 (13.71, 21.39)	
		Pre-Vax	17.37 (12.98, 21.76)		17.56 (12.59, 22.53)		18.07 (14.07, 22.06)	
		Wk6	15.10 (12.54, 17.66)	0.9011	15.15 (11.58, 18.72)	0.3385	14.60 (10.87, 18.33)	0.4208
		Wk22	11.60 (9.19, 14.01)	0.1400	16.97 (6.37, 27.57)	0.9997	12.56 (8.93, 16.18)	0.0805
		Wk28	16.89 (13.87, 19.91)	0.9992	14.35 (9.37, 19.33)	0.7109	12.88 (9.61, 16.16)	**0.0336**
	CD8+ Central Memory T cells	Pre-PBio	12.49 (9.43, 15.55)		10.66 (8.25, 13.06)		13.53 (9.65, 17.40)	
		Pre-Vax	12.71 (7.77, 17.64)		11.04 (7.59, 14.48)		13.15 (9.81, 16.50)	
		Wk6	12.59 (8.61, 16.56)	>0.9999	13.21 (9.89, 16.53)	0.5653	14.08 (11.00, 17.16)	0.9951
		Wk22	11.02 (7.90, 14.14)	0.7833	7.25 (2.95, 11.55)	0.3571	10.74 (5.97, 15.51)	0.9407
		Wk28	12.91 (7.38, 17.01)	0.9948	10.70 (6.58, 14.82)	0.9999	12.35 (6.78, 17.92)	0.9990
	CD8+ Effector Memory T cells	Pre-PBio	15.04 (12.34, 17.74)		14.17 (12.47, 15.88)		13.96 (11.17, 16.74)	
		Pre-Vax	14.48 (11.12, 17.84)		16.67 (12.55, 20.80)		15.02 (12.49, 17.54)	
		Wk6	13.10 (9.65, 16.54)	0.9083	10.96 (7.40, 14.51)	**0.0271**	11.91 (8.65, 15.17)	0.2823
		Wk22	11.55 (8.56, 14.55)	0.6655	15.20 (7.07, 23.34)	0.9869	12.52 (8.73, 16.31)	0.7286
		Wk28	11.91 (9.02, 14.80)	0.6122	13.81 (9.54, 18.09)	0.8302	12.58 (8.65, 16.51)	0.7067
Colon	CD8+ T cells	Pre-PBio	22.03 (17.58, 26.48)		26.48 (18.70, 28.34)		21.86 (16.34, 27.38)	
		Pre-Vax	18.76 (14.94, 22.58)		22.45 (17.56, 27.34)		23.81 (17.56, 30.06)	
		Wk6	16.27 (12.93, 19.60)	0.4486	22.40 (16.88, 27.92)	>0.9999	17.97 (14.21, 21.73)	0.1775
		Wk22	16.27 (13.08, 19.47)	0.6309	19.12 (12.18, 26.06)	0.7437	14.98 (12.19, 17.77)	0.0853
		Wk28	20.59 (17.04, 24.14)	0.9282	18.52 (13.78, 23.26)	0.6147	20.20 (14.32, 26.08)	0.6577
	CD8+ Central Memory T cells	Pre-PBio	12.04 (8.46, 15.62)		9.78 (7.58, 11.97)		13.95 (10.97, 16.93)	
		Pre-Vax	14.05 (11.76, 16.34)		9.73 (6.82, 12.65)		12.84 (9.83, 15.85)	
		Wk6	15.53 (10.96, 20.10)	0.9478	12.70 (9.90, 15.49)	0.1977	17.69 (13.09, 22.29)	0.3219
		Wk22	12.67 (7.51, 17.82)	0.9667	11.08 (5.77, 16.39)	0.9364	14.33 (8.47, 20.19)	0.9879
		Wk28	11.44 (8.15, 14.72)	0.3343	9.72 (6.14, 13.29)	>0.9999	12.88 (8.60, 17.16)	>0.9999
	CD8+ Effector Memory T cells	Pre-PBio	13.74 (10.98, 16.50)		17.01 (13.29, 20.72)		12.08 (8.84, 15.31)	
		Pre-Vax	16.16 (12.57, 19.75)		18.69 (14.34, 23.04)		16.91 (11.46, 22.35)	
		Wk6	9.89 (6.88, 12.91)	0.1133	15.30 (11.69, 18.91)	0.3119	10.53 (7.42, 13.64)	**0.0196**
		Wk22	13.05 (8.68, 17.42)	0.7957	15.78 (8.76, 22.79)	0.6270	10.25 (5.22, 15.29)	0.1296
		Wk28	13.66 (10.40, 16.93)	0.8552	14.30 (10.67, 17.93)	0.1454	12.12 (8.43, 15.81)	0.3456

[1]CI: Confidence Interval.

[2]Multiplicity adjusted p values indicating the statistical significance between the Pre-Vax time point and subsequent time points. Statistical significance calculated using a two-way repeated measures mixed-effects model with the Geisser-Greenhouse correction and a Tukey's multiple comparisons test, with individual variances computed for each comparison.

Supplementary Table 6. Mean (95% CI[1]) and p values for statistical analysis of differences in %Ki-67+ of CD8+ T cell subset frequencies between Pre-Vax and subsequent time points in rectum and colon tissue

			Probiotics+Vaccine		Vaccine only		Probiotics only	
			**Mean (95% CI[1])**	**p value[3,5]**	**Mean (95% CI[1])**	**p value[3,5]**	**Mean (95% CI[1])**	**p value[3,5]**
**Rectum**	**%Ki-67+ of CD8+ T cells**	**Pre-PBio**	8.78 (4.56, 13.00)		9.21 (5.68, 12.74)		7.85 (5.02, 10.67)	
		**Pre-Vax**	8.91 (5.95, 11.86)		8.81 (6.87, 10.74)		7.98 (6.05, 9.91)	
		**Wk6**	11.33 (7.87, 14.79)	0.5448	11.22 (6.08, 16.35)	0.8798	8.48 (6.20, 10.76)	0.9965
		**Wk22**	13.87 (8.48, 19.26)	0.4457	17.03 (8.44, 25.65)	0.1049	12.29 (7.12, 17.46)	0.3683
		**Wk28**	9.37 (6.84, 11.89)	0.9961	10.90 (7.15, 14.64)	0.6964	8.71 (6.71, 10.72)	0.9284
	**%Ki-67+ of CD8+ Central Memory T cells**	**Pre-PBio**	9.90 (7.41, 12.38)		10.55 (8.32, 12.78)		10.69 (8.27, 13.11)	
		**Pre-Vax**	12.12 (9.15, 15.06)		8.65 (6.33, 10.97)		9.84 (8.48, 11.20)	
		**Wk6**	12.22 (8.30, 16.13)	>0.9999	10.94 (7.77, 14.10)	0.7048	9.94 (7.67, 12.21)	>0.9999
		**Wk22**	13.62 (11.21, 16.03)	0.7886	15.47 (8.69, 22.24)	**0.0340**	12.90 (9.01, 16.78)	0.5920
		**Wk28**	12.61 (9.33, 15.89)	0.9965	9.73 (5.86, 13.59)	0.8743	12.51 (6.51, 18.50)	0.8800
	**%Ki-67+ of CD8+ Effector Memory T cells**	**Pre-PBio**	9.08 (4.69, 13.48)		9.04 (6.17, 11.92)		7.89 (4.87, 10.91)	
		**Pre-Vax**	7.98 (4.47, 11.48)		8.70 (5.52, 11.87)		7.79 (4.96, 10.62)	
		**Wk6**	11.14 (8.59, 13.70)	0.3960	9.99 (5.99, 13.98)	0.9700	9.57 (6.53, 12.61)	0.8425
		**Wk22**	11.19 (7.38, 15.00)	0.5889	12.11 (4.96, 19.25)	0.2726	12.72 (9.93, 15.51)	0.0745
		**Wk28**	8.50 (6.35, 10.66)	0.9903	8.60 (5.34, 11.86)	>0.9999	8.17 (6.22, 10.11)	0.9937
**Colon**	**%Ki-67+ of CD8+ T cells**	**Pre-PBio**	8.72 (3.93, 13.51)		7.44 (3.29, 11.59)		7.09 (4.19, 9.98)	
		**Pre-Vax**	7.88 (5.74, 10.03)		8.06 (5.56, 10.55)		8.03 (6.16, 9.90)	
		**Wk6**	8.26 (5.68, 10.84)	0.9993	7.64 (4.90, 10.38)	0.9969	5.68 (3.36, 8.00)	0001211
		**Wk22**	10.90 (6.45, 15.35)	0.7023	11.05 (4.81, 17.29)	0.7192	8.50 (4.80, 12.19)	0.9981
		**Wk28**	8.50 (6.76, 10.24)	0.9393	7.29 (4.67, 9.92)	0.9795	6.15 (4.72, 7.57)	0.4455
	**%Ki-67+ of CD8+ Central Memory T cells**	**Pre-PBio**	9.34 (6.73, 11.96)		9.52 (7.66, 11.37)		9.16 (7.36, 10.96)	
		**Pre-Vax**	8.04 (6.38, 9.69)		11.06 (8.90, 13.22)		9.78 (7.61, 11.95)	
		**Wk6**	9.31 (7.34, 11.28)	0.8944	8.52 (5.33, 11.71)	0.5596	7.29 (4.71, 9.89)	0.5199
		**Wk22**	11.06 (8.23, 13.88)	0.1165	9.98 (7.02, 12.95)	0.9686	10.21 (7.96, 12.46)	0.9977
		**Wk28**	13.58 (8.78, 18.37)	0.1057	10.54 (8.75, 12.33)	0.9865	8.85 (6.32, 11.38)	0.9453
	**%Ki-67+ of CD8+ Effector Memory T cells**	**Pre-PBio**	8.69 (4.16, 13.23)		8.17 (3.96, 12.37)		7.07 (3.79, 10.34)	
		**Pre-Vax**	7.70 (5.09, 10.31)		8.06 (4.55, 11.58)		8.01 (6.29, 9.72)	
		**Wk6**	7.96 (5.35, 10.56)	0.9999	6.20 (3.86, 8.53)	0.7638	6.16 (4.14, 8.18)	0.2297
		**Wk22**	8.80 (5.79, 11.80)	0.9699	9.35 (2.13, 16.56)	0.9946	9.08 (7.16, 11.01)	0.5418
		**Wk28**	9.07 (5.95, 12.20)	0.8769	9.19 (5.10, 13.29)	0.9787	9.01 (4.12, 7.90)	0.3733

[1]CI: Confidence Interval.

[2]Multiplicity adjusted p values indicating the statistical significance between the Pre-Vax time point and subsequent time points. Statistical significance calculated using a two-way repeated measures mixed-effects model with the Geisser-Greenhouse correction and a Tukey's multiple comparisons test, with individual variances computed for each comparison.

Supplementary Table 7. Mean (95% CI[1]) and p values for statistical analysis of differences in %cytokine expressing CD4+ and CD8+ T cell subset frequencies between Pre-Vax and subsequent time points in colon tissue

			Probiotics+Vaccine		Vaccine only		Probiotics only	
			Mean (95% CI[1])	p value[3,5]	Mean (95% CI[1])	p value[3,5]	Mean (95% CI[1])	p value[3,5]
Colon	%TNF-a+ of CD4+ T cells	Pre-PBio	40.51 (34.99, 46.02)		43.30 (36.45, 50.15)		43.52 (36.90, 50.14)	
		Pre-Vax	33.69 (21.79, 45.58)		30.95 (17.45, 44.45)		37.25 (22.12, 52.38)	
		Wk6	26.36 (15.96, 36.76)	0.0968	29.44 (15.97, 42.91)	0.9213	23.20 (10.23, 36.17)	0.0551
		Wk22	41.59 (21.99, 61.20)	0.8159	38.48 (19.51, 57.46)	0.8891	26.21 (8.84, 43.57)	0.5924
		Wk28	27.52 (10.02, 45.02)	0.9625	25.23 (11.92, 38.55)	0.9687	22.43 (4.80, 40.07)	0.6646
	%IFN-g+ of CD4+ T cells	Pre-PBio	14.97 (10.29, 19.65)		19.44 (14.61, 24.28)		13.92 (10.47, 17.37)	
		Pre-Vax	12.61 (7.77, 17.44)		11.95 (6.82, 17.08)		13.24 (6.86, 19.62)	
		Wk6	12.84 (7.47, 18.22)	>0.9999	15.36 (9.10, 21.62)	0.6905	10.04 (3.78, 16.30)	0.6279
		Wk22	18.10 (7.33, 28.86)	0.7073	17.32 (7.69, 26.94)	0.3994	9.08 (3.55, 14.60)	0.6642
		Wk28	8.59 (3.81, 13.36)	0.5182	9.36 (4.09, 14.63)	0.8555	7.22 (0.90, 13.55)	0.3616
	%IL-10+ of CD4+ T cells	Pre-PBio	12.03 (8.95, 15.12)		16.05 (11.89, 20.22)		14.98 (11.32, 18.64)	
		Pre-Vax	12.75 (9.41, 16.10)		15.69 (10.10, 21.28)		12.47 (8.75, 16.19)	
		Wk6	12.38 (7.90, 16.85)	0.9996	17.99 (11.64, 24.35)	0.9356	10.22 (6.26, 14.18)	0.7902
		Wk22	7.58 (5.26, 9.89)	0.1099	12.68 (8.54, 16.81)	0.7969	8.38 (3.63, 13.12)	0.7048
		Wk28	12.34 (6.91, 17.76)	0.9996	12.81 (6.65, 18.97)	0.9246	10.46 (5.72, 15.19)	0.9533
	%IL-17A+ of CD4+ T cells	Pre-PBio	24.31 (19.91, 28.70)		29.36 (22.54, 36.18)		28.47 (23.62, 33.32)	
		Pre-Vax	23.83 (17.58, 30.08)		24.55 (16.28, 32.82)		23.41 (16.82, 30.00)	
		Wk6	23.37 (15.71, 31.03)	0.9997	30.19 (21.01, 39.37)	0.6976	19.35 (13.68, 25.02)	0.6967
		Wk22	17.19 (12.60, 21.79)	0.2501	22.22 (17.41, 27.03)	0.9455	15.55 (8.67, 22.44)	0.5031
		Wk28	21.32 (15.64, 27.00)	0.9460	21.47 (12.87, 30.08)	0.9819	20.07 (13.28, 26.86)	0.9604
	%IFN-g+ of CD8+ T cells	Pre-PBio	15.02 (11.70, 18.34)		16.61 (8.47, 24.76)		16.98 (9.15, 24.81)	
		Pre-Vax	13.33 (8.14, 18.51)		10.06 (5.93, 14.19)		11.40 (7.65, 15.15)	
		Wk6	15.73 (8.24, 23.21)	0.9529	17.84 (6.94, 28.74)	0.4413	15.21 (5.31, 25.10)	0.8137
		Wk22	16.54 (4.00, 29.08)	0.9299	12.91 (6.02, 19.79)	0.8610	10.60 (5.08, 16.13)	0.9966
		Wk28	7.43 (4.56, 10.30)	0.2529	7.85 (4.36, 11.34)	0.9400	7.35 (2.92, 11.78)	0.4301
	%IL-22+ of CD8+ T cells	Pre-PBio	10.96 (8.82, 13.10)		10.83 (8.14, 13.51)		13.60 (9.87, 17.34)	
		Pre-Vax	11.18 (8.75, 13.60)		8.12 (6.55, 9.70)		12.28 (9.24, 15.31)	
		Wk6	17.87 (2.70, 33.03)	0.8535	12.93 (6.30, 19.56)	0.5536	12.81 (3.06, 22.55)	0.9998
		Wk22	11.44 (7.61, 15.26)	0.9998	11.48 (8.19, 14.77)	0.1039	9.55 (4.85, 14.25)	0.8668
		Wk28	9.27 (5.85, 12.69)	0.9143	8.08 (5.59, 10.56)	>0.9999	8.77 (5.90, 11.63)	0.4624

[1]CI: Confidence Interval.

[2]Multiplicity adjusted p values indicating the statistical significance between the Pre-Vax time point and subsequent time points. Statistical significance calculated using a two-way repeated measures mixed-effects model with the Geisser-Greenhouse correction and a Tukey's multiple comparisons test, with individual variances computed for each comparison.

Supplementary Table 8. Mean (95% CI[1]) and p values for statistical analysis of differences in total peptide-specific CD4+ and CD8+ T cell responses to HIV env and SIV gag between experimental groups in PBMCs

			Probiotics+ Vaccine	Vaccine only	Probiotics only	Probiotics+Vaccine vs. Vaccine only	Probiotics+Vaccine vs. Probiotics only	Vaccine only vs. Probiotics only
			Mean (95% CI[1])	Mean (95% CI[1])	Mean (95% CI[1])	p value[2]	p value[2]	p value[2]
PBMC	HIV env CD4+	Pre-Vax	0.04 (0.02, 0.06)	0.13 (0.05, 0.22)	0.16 (-0.03, 0.35)	0.1633	>0.9999	0.9247
		Wk28	0.08 (0.03, 0.13)	0.19 (0.02, 0.35)	0.06 (0.02, 0.10)	0.6917	>0.9999	0.2045
	HIV env CD8+	Pre-Vax	0.11 (0.003, 0.22)	0.20 (0.04, 0.35)	0.15 (0.01, 0.28)	0.3594	>0.9999	0.6068
		Wk28	0.13 (0.03, 0.23)	0.17 (0.04, 0.29)	0.13 (0.01, 0.24)	>0.9999	>0.9999	0.8540
	SIV gag CD4+	Pre-Vax	0.04 (0.02, 0.06)	0.16 (0.06, 0.26)	0.28 (0.02, 0.54)	0.1084	0.0761	>0.9999
		Wk28	0.23 (0.04, 0.41)	0.18 (0.07, 0.29)	0.13 (0.06, 0.21)	>0.9999	0.8737	>0.9999
	SIV gag CD8+	Pre-Vax	0.08 (0.01, 0.15)	0.32 (0.14, 0.49)	0.39 (0.02, 0.76)	0.0063	0.1044	>0.9999
		Wk28	0.48 (0.12, 0.84)	0.35 (0.13, 0.56)	0.31 (0.08, 0.53)	>0.9999	>0.9999	>0.9999

[1]CI: Confidence Interval

[2]Multiplicity adjusted p values indicating the statistical significance between the indicated experimental groups at each time point, calculated using a Kruskal-Wallis test with Dunn's multiple comparisons test.

Supplementary Table 9. Mean (95% CI[1]) and p values for statistical analysis of differences in individual peptide-specific CD4+ and CD8+ T cell responses to HIV env and SIV gag between experimental groups in PBMCs and jejunum tissue

				Probiotics+Vaccine	Vaccine only	Probiotics only	Probiotics+ Vaccine vs. Vaccine only	Probiotics+ Vaccine vs. Probiotics only	Vaccine only vs. Probiotics only
				Mean (95% CI[1])	Mean (95% CI[1])	Mean (95% CI[1])	p value[2]	p value[2]	p value[2]
PBMC	HIV env CD4+	IFN-g	Pre-Vax	0.01 (0.004, 0.02)	0.02 (0.01, 0.03)	0.03 (-0.01, 0.06)	0.9801	0.8954	0.9642
			Wk28	0.01 (0.005, 0.01)	0.02 (0.01, 0.03)	0.01 (0.004, 0.02)	0.9315	0.9989	0.9142
		IL-2	Pre-Vax	0.01 (0.003, 0.02)	0.02 (0.004, 0.03)	0.04 (-0.01, 0.09)	0.9914	0.7903	0.8572
			Wk28	0.03 (0.01, 0.04)	0.06 (0.02, 0.11)	0.04 (0.001, 0.07)	0.4245	0.9452	0.6201
		TNF-a	Pre-Vax	0.02 (0.002, 0.03)	0.01 (0.002, 0.03)	0.03 (-0.01, 0.07)	0.9998	0.8946	0.8862
			Wk28	0.03 (0.004, 0.06)	0.07 (0.02, 0.13)	0.02 (0.004, 0.03)	0.2831	0.9201	0.1396
		CD107 a+Grz B+	Pre-Vax	0.01 (-0.001, 0.03)	0.10 (0.01, 0.18)	0.11 (-0.06, 0.28)	0.0797	0.0388	0.9511
			Wk28	0.04 (-0.02, 0.09)	0.08 (-0.03, 0.20)	0.01 (0.01, 0.02)	0.2439	0.6388	0.0351
	HIV env CD8+	IFN-g	Pre-Vax	0.03 (0.01, 0.06)	0.05 (0.03, 0.07)	0.03 (0.01, 0.05)	0.9102	0.9770	0.8100
			Wk28	0.03 (0.01, 0.06)	0.04 (0.01, 0.06)	0.04 (0.01, 0.06)	0.9997	0.9988	0.9997
		IL-2	Pre-Vax	0.02 (0.002, 0.03)	0.02 (0.001, 0.04)	0.04 (-0.001, 0.07)	0.9991	0.9011	0.9174
			Wk28	0.04 (0.01, 0.07)	0.04 (0.01, 0.07)	0.04 (0.01, 0.07)	0.9933	0.9966	0.9995
		TNF-a	Pre-Vax	0.05 (-0.03, 0.13)	0.06 (0.02, 0.10)	0.05 (-0.003, 0.11)	0.9954	>0.9999	0.9955
			Wk28	0.03 (0.001, 0.05)	0.03 (0.01, 0.05)	0.04 (0.01, 0.07)	0.9918	0.9570	0.9860
		CD107a+ GrzB+	Pre-Vax	0.07 (-0.02, 0.16)	0.14 (-0.01, 0.28)	0.08 (-0.03, 0.18)	0.2608	0.9869	0.3340
			Wk28	0.09 (-0.02, 0.19)	0.11 (-0.02, 0.23)	0.06 (-0.04, 0.16)	0.8484	0.7839	0.4485
	SIV gag CD4+	IFN-g	Pre-Vax	0.01 (0.01, 0.02)	0.02 (0.01, 0.04)	0.01 (0.002, 0.01)	0.9863	0.9934	0.9612
			Wk28	0.01 (0.01, 0.02)	0.01 (0.01, 0.02)	0.01 (-0.003, 0.02)	0.9993	0.9987	0.9961
		IL-2	Pre-Vax	0.01 (0.004, 0.02)	0.04 (-0.003, 0.09)	0.03 (-0.001, 0.05)	0.8649	0.9713	0.9567
			Wk28	0.15 (-0.04, 0.34)	0.09 (0.02, 0.15)	0.06 (0.02, 0.09)	0.3768	0.1182	0.7891
		TNF-a	Pre-Vax	0.01 (0.004, 0.01)	0.04 (-0.01, 0.08)	0.02 (-0.01, 0.05)	0.8276	0.9702	0.9339
			Wk28	0.05 (0.02, 0.07)	0.06 (-0.0003, 0.11)	0.02 (0.004, 0.04)	0.9845	0.8461	0.7526
		CD107a+ GrzB+	Pre-Vax	0.02 (-0.001, 0.03)	0.09 (0.01, 0.18)	0.26 (-0.01, 0.53)	0.3236	<0.0001	0.0054
			Wk28	0.12 (-0.0001, 0.25)	0.09 (0.02, 0.15)	0.08 (0.02, 0.15)	0.7147	0.6920	0.9992
	SIV gag CD8+	IFN-g	Pre-Vax	0.02 (0.01, 0.04)	0.05 (0.02, 0.08)	0.03 (0.01, 0.06)	0.9065	0.9931	0.9484
			Wk28	0.03 (0.01, 0.05)	0.03 (0.01, 0.04)	0.04 (0.003, 0.07)	0.9997	0.9980	0.9961
		IL-2	Pre-Vax	0.02 (0.004, 0.04)	0.06 (0.02, 0.09)	0.04 (0.01, 0.07)	0.8942	0.9800	0.9637
			Wk28	0.24 (-0.11, 0.60)	0.09 (-0.003, 0.18)	0.09 (0.02, 0.16)	0.3249	0.3170	0.9999
		TNF-a	Pre-Vax	0.02 (0.003, 0.04)	0.11 (-0.01, 0.23)	0.05 (-0.01, 0.11)	0.1361	0.9175	0.7150
			Wk28	0.06 (0.01, 0.12)	0.13 (0.03, 0.22)	0.07 (0.02, 0.11)	0.8306	0.9994	0.8475
		CD107a+ GrzB+	Pre-Vax	0.04 (-0.01, 0.09)	0.20 (0.07, 0.33)	0.34 (-0.01, 0.68)	0.0832	0.0003	0.1416
			Wk28	0.37 (0.05, 0.69)	0.23 (0.02, 0.43)	0.23 (0.02, 0.43)	0.3510	0.3601	0.9998

Jejunum	HIV env CD4+	IFN-g	**Pre-Vax**	0.11 (-0.04, 0.26)	0.16 (-0.12, 0.44)	0.19 (-0.07, 0.45)	0.8624	0.6436	0.9303
			**Wk28**	0.04 (-0.05, 0.14)	0.13 (-0.04, 0.31)	0.06 (-0.02, 0.14)	0.3110	0.9718	0.4042
		IL-2	**Pre-Vax**	0.02 (0.001, 0.04)	0.07 (-0.04, 0.18)	0.12 (-0.01, 0.24)	0.8434	0.5337	0.8719
			**Wk28**	0.05 (-0.01, 0.10)	0.15 (-0.02, 0.31)	0.03 (-0.03, 0.09)	0.2275	0.9703	0.1332
		TNF-a	**Pre-Vax**	0.02 (-0.01, 0.04)	0.09 (-0.05, 0.22)	0.01 (-0.01, 0.03)	0.7624	0.9970	0.7042
			**Wk28**	0.08 (-0.05, 0.22)	0.02 (-0.01, 0.06)	0.02 (-0.01, 0.06)	0.5722	0.5847	0.9984
	HIV env CD8+	IFN-g	**Pre-Vax**	0.07 (-0.03, 0.17)	0.17 (-0.14, 0.48)	0.12 (-0.02, 0.27)	0.3897	0.7490	0.8000
			**Wk28**	0.04 (-0.03, 0.12)	0.07 (-0.01, 0.19)	0.06 (-0.05, 0.18)	0.9265	0.9441	0.9979
		IL-2	**Pre-Vax**	0.04 (-0.02, 0.10)	0.09 (-0.04, 0.22)	0.04 (-0.01, 0.10)	0.7915	0.9972	0.8189
			**Wk28**	0.02 (-0.01, 0.06)	0.09 (-0.01, 0.19)	0.01 (-0.004, 0.02)	0.4654	0.9752	0.3274
		TNF-a	**Pre-Vax**	0.03 (-0.02, 0.07)	0.05 (-0.03, 0.14)	0.02 (-0.02, 0.06)	0.9282	0.9935	0.8769
			**Wk28**	0.13 (-0.08, 0.34)	0.04 (-0.01, 0.09)	0.01 (-0.01, 0.04)	0.2730	0.1086	0.8955
	SIV gag CD4+	IFN-g	**Pre-Vax**	0.04 (-0.02, 0.10)	0.16 (-0.04, 0.37)	0.36 (0.04, 0.69)	0.3666	0.0015	0.0696
			**Wk28**	0.10 (-0.05, 0.24)	0.03 (-0.03, 0.09)	0.06 (-0.01, 0.14)	0.5351	0.8499	0.8400
		IL-2	**Pre-Vax**	0.03 (-0.02, 0.08)	0.12 (-0.002, 0.24)	0.08 (-0.02, 0.19)	0.6058	0.8435	0.9019
			**Wk28**	0.11 (0.03, 0.20)	0.07 (-0.05, 0.18)	0.03 (-0.01, 0.07)	0.7625	0.3631	0.7965
		TNF-a	**Pre-Vax**	0.04 (0.01, 0.08)	0.09 (0.02, 0.16)	0.01 (-0.02, 0.04)	0.8529	0.9463	0.6598
			**Wk28**	0.13 (-0.06, 0.32)	0.06 (-0.05, 0.17)	0.05 (-0.03, 0.13)	0.4666	0.3895	0.949
	SIV gag CD8+	IFN-g	**Pre-Vax**	0.03 (-0.02, 0.08)	0.12 (-0.03, 0.28)	0.12 (-0.05, 0.28)	0.2877	0.3205	0.9922
			**Wk28**	0.03 (-0.01, 0.07)	0.003 (-0.005, 0.01)	0.02 (-0.01, 0.05)	0.9388	0.9940	0.9674
		IL-2	**Pre-Vax**	0.03 (-0.01, 0.06)	0.07 (-0.03, 0.16)	0.01 (-0.01, 0.04)	0.7802	0.9709	0.6260
			**Wk28**	0.06 (-0.01, 0.13)	0.09 (0.002, 0.17)	0.06 (0.02, 0.10)	0.9054	>0.9999	0.8998
		TNF-a	**Pre-Vax**	0.08 (0.02, 0.14)	0.04 (-0.02, 0.09)	0.07 (-0.06, 0.19)	0.7073	0.9518	0.8609
			**Wk28**	0.25 (-0.10, 0.59)	0.05 (-0.02, 0.11)	0.01 (-0.02, 0.04)	0.0157	0.0032	0.8867

[1]CI: Confidence Interval
[2]Multiplicity adjusted p values indicating the statistical significance between the indicated experimental groups at each time point, calculated using a two-way ANOVA and Tukey's multiple comparisons test, with individual variances computed for each comparison.

Supplementary Table 10. Mean (95% CI[1]) and p values for statistical analysis of differences in B cell frequencies between Pre-Vax and subsequent time points in rectum and colon tissue

			Probiotics+Vaccine		Vaccine only		Probiotics only	
			Mean (95% CI[1])	p value[2]	Mean (95% CI[1])	p value[2]	Mean (95% CI[1])	p value[2]
Rectum	B cells	Pre-PBio	22.50 (15.99, 29.01)		24.29 (13.23, 35.35)		18.80 (9.96, 27.65)	
		Pre-Vax	27.44 (17.87, 37.01)		27.99 (16.87, 39.11)		21.28 (13.63, 28.93)	
		Wk6	28.05 (17.31, 38.79)	>0.9999	25.12 (13.08, 37.16)	0.9010	32.18 (19.83, 44.53)	0.3421
		Wk22	40.80 (28.39, 53.21)	0.3306	25.34 (11.32, 39.35)	0.9861	28.86 (27.01, 50.70)	**0.0229**
		Wk28	23.97 (17.16, 30.78)	0.9229	23.48 (11.90, 35.07)	0.9616	29.67 (15.72, 43.62)	0.4037
	%IgA+ of B cells	Pre-PBio	33.72 (25.72, 41.72)		30.69 (23.21, 38.16)		31.59 (25.89, 37.29)	
		Pre-Vax	24.94 (19.62, 30.26)		30.99 (22.79, 39.19)		29.38 (21.00, 37.76)	
		Wk6	28.60 (20.79, 36.41)	0.7012	25.85 (19.90, 31.80)	0.8417	27.57 (20.80, 34.35)	0.9919
		Wk22	33.94 (24.34, 43.55)	0.1636	25.61 (18.38, 32.84)	0.3225	35.24 (26.92, 43.57)	0.8026
		Wk28	27.65 (19.67, 35.63)	0.9368	27.12 (22.03, 32.21)	0.8243	29.12 (21.75, 36.49)	>0.9999
	%IgG+ of B cells	Pre-PBio	10.77 (6.13, 15.41)		9.25 (5.87, 12.62)		9.87 (6.59, 13.15)	
		Pre-Vax	11.00 (5.56, 16.44)		9.97 (2.87, 17.06)		12.70 (5.42, 19.98)	
		Wk6	12.18 (8.00, 16.35)	0.9663	10.66 (7.71, 13.60)	0.9979	8.20 (5.91, 10.48)	0.4756
		Wk22	7.77 (4.08, 11.47)	0.3526	9.58 (5.82, 13.35)	0.9998	7.29 (3.78, 10.81)	0.3325
		Wk28	11.02 (5.88, 16.17)	>0.9999	11.02 (8.21, 13.84)	0.9964	9.09 (4.93, 13.24)	0.5179
Colon	B cells	Pre-PBio	14.42 (8.53, 20.31)		10.19 (6.49, 13.88)		11.80 (7.82, 15.77)	
		Pre-Vax	18.14 (9.16, 27.12)		16.34 (6.88, 25.79)		12.44 (5.90, 18.98)	
		Wk6	24.14 (12.34, 35.93)	0.7090	9.18 (4.70, 13.65)	0.2159	23.44 (14.45, 32.42)	0.1305
		Wk22	24.02 (15.02, 33.01)	0.5832	16.33 (9.37, 23.30)	>0.9999	29.23 (19.23, 39.23)	0.0686
		Wk28	16.01 (6.35, 25.67)	0.9732	17.81 (9.72, 25.90)	0.9978	15.86 (7.27, 24.44)	0.9358
	%IgA+ of B cells	Pre-PBio	43.82 (38.30, 49.34)		39.22 (30.59, 47.85)		35.22 (28.54, 41.89	
		Pre-Vax	41.34 (33.94, 48.74)		31.01 (25.27, 36.75)		30.58 (24.08, 37.08)	
		Wk6	39.60 (31.08, 48.12)	0.9812	40.29 (32.13, 48.45)	0.1266	45.18 (34.71, 55.65)	0.0578
		Wk22	40.43 (30.83, 50.03)	0.9998	44.28 (33.67, 54.89)	0.0588	42.53 (33.85, 51.21)	0.1612
		Wk28	33.72 (24.10, 43.34)	0.7326	32.89 (26.29, 39.49)	0.9897	34.95 (27.66, 42.24)	0.8655
	%IgG+ of B cells	Pre-PBio	7.62 (4.71, 10.53)		7.93 (5.36, 10.50)		8.36 (3.90, 12.83)	
		Pre-Vax	7.34 (4.27, 10.40)		8.36 (4.91, 11.80)		7.95 (4.16, 11.75)	
		Wk6	6.53 (4.21, 8.84)	0.9328	7.69 (4.74, 10.64)	0.9864	5.83 (2.69, 8.98)	0.7864
		Wk22	6.67 (3.58, 9.76)	0.9953	5.65 (2.48, 8.82)	0.6652	4.78 (3.07, 6.49)	0.4084
		Wk28	12.43 (7.54, 17.33)	0.2582	7.60 (3.37, 11.83)	0.9970	7.50 (4.66, 10.34)	0.9983

[1]CI: Confidence Interval.

[2]Multiplicity adjusted p values indicating the statistical significance between the Pre-Vax time point and subsequent time points. Statistical significance calculated using a two-way repeated measures mixed-effects model with the Geisser-Greenhouse correction and a Tukey's multiple comparisons test, with individual variances computed for each comparison.

Supplementary Table 11. MHC Genotype

Animal Number	Group	*Mamu-A*01*	*Mamu-B*08*	*Mamu-B*17*
Z08113	Probiotics+Vaccine	–	–	–
Z12060	Probiotics+Vaccine	–	–	–
Z08065	Probiotics+Vaccine	–	–	+
A16254	Probiotics+Vaccine	–	–	–
A16258	Probiotics+Vaccine	–	–	–
A16261	Probiotics+Vaccine	–	–	–
A17119	Probiotics+Vaccine	–	+	–
A17123	Probiotics+Vaccine	–	–	–
A17244	Probiotics+Vaccine	–	–	–
A17245	Probiotics+Vaccine	–	–	+
Z08116	Vaccine only	–	–	–
Z10071	Vaccine only	–	–	–
Z11349	Vaccine only	–	–	–
A16251	Vaccine only	+	–	–
A16252	Vaccine only	–	–	–
A16253	Vaccine only	–	–	–
A17122	Vaccine only	–	–	–
A17118	Vaccine only	–	–	+
A17124	Vaccine only	–	–	–
A17239	Vaccine only	+	–	–
Z08134	Probiotics only	+	–	+
Z08114	Probiotics only	–	–	–
A16256	Probiotics only	–	–	–
A16260	Probiotics only	–	–	–
A16259	Probiotics only	–	–	+
A17125	Probiotics only	–	+	–
A17120	Probiotics only	+	–	–
A17242	Probiotics only	–	–	+
A17243	Probiotics only	–	–	–
A17246	Probiotics only	–	–	–
A16263	Control	–	–	+
A16250	Control	+	–	–
A16257	Control	–	–	–
A16262	Control	–	+	–
Z11348	Control	–	–	–
A17126	Control	–	–	–
A17121	Control	–	–	–
A17238	Control	+	–	–
Z11089	Control	–	–	–
A17241	Control	–	–	+
A17240	Control	–	–	–

MHC allele expression in rhesus macaques treated with probiotics+vaccine (n=10), vaccine only (n=10), probiotics only (n=10) and no probiotics/no vaccine controls (n=11). Positive expression of the indicated allele: +. No expression of the indicated allele: –.
