# Supplementary material for: Effects of persistent modulation of intestinal microbiota on SIV/HIV vaccination in rhesus macaques
Source: NPJ Vaccines. 2021 Mar 11;6:34. doi: 10.1038/s41541-021-00298-4 (PMC7952719; doi:10.1038/s41541-021-00298-4)
Supplement: Supplementary file 2 — Reporting Summary [file 41541_2021_298_MOESM2_ESM.pdf]

nature research

Corresponding author(s): Nichole R. Klatt, Jennifer A. Manuzak

Last updated by author(s): Feb 4, 2021

Reporting Summary

Nature Research wishes to improve the reproducibility of the work that we publish. This form provides structure for consistency and transparency in reporting. For further information on Nature Research policies, see our Editorial Policies and the Editorial Policy Checklist.

Statistics

For all statistical analyses, confirm that the following items are present in the figure legend, table legend, main text, or Methods section.

n/a	Confirmed	
☐	☒	The exact sample size (*n*) for each experimental group/condition, given as a discrete number and unit of measurement
☒	☐	A statement on whether measurements were taken from distinct samples or whether the same sample was measured repeatedly
☐	☒	The statistical test(s) used AND whether they are one- or two-sided<br>*Only common tests should be described solely by name; describe more complex techniques in the Methods section.*
☒	☐	A description of all covariates tested
☐	☒	A description of any assumptions or corrections, such as tests of normality and adjustment for multiple comparisons
☐	☒	A full description of the statistical parameters including central tendency (e.g. means) or other basic estimates (e.g. regression coefficient) AND variation (e.g. standard deviation) or associated estimates of uncertainty (e.g. confidence intervals)
☒	☐	For null hypothesis testing, the test statistic (e.g. *F*, *t*, *r*) with confidence intervals, effect sizes, degrees of freedom and *P* value noted<br>*Give P values as exact values whenever suitable.*
☒	☐	For Bayesian analysis, information on the choice of priors and Markov chain Monte Carlo settings
☒	☐	For hierarchical and complex designs, identification of the appropriate level for tests and full reporting of outcomes
☐	☒	Estimates of effect sizes (e.g. Cohen's *d*, Pearson's *r*), indicating how they were calculated

Our web collection on statistics for biologists contains articles on many of the points above.

Software and code

Policy information about availability of computer code

Data collection	MiSeq Control Software, including MiSeq Reporter (version 3.1)<br>FACS Diva (Version 8)
Data analysis	R studio (version 1.2.5033), Microbiome package (version 1.8.0), Vegan package for Ordination Diversity and Dissimilarities (version 2.5-6), Pairwise Adonis package (version 0.0.1), FSA package (version 0.8.30), DESeq2 (version 1.26.0), phyloseq package (version 1.30.0), ggplot package (version 3.2.1).<br>QIIME2 (version 2019.4)<br>FlowJo (Version 9.9.6 or 10.0.8)<br>GraphPad Prism (Version 8)

For manuscripts utilizing custom algorithms or software that are central to the research but not yet described in published literature, software must be made available to editors and reviewers. We strongly encourage code deposition in a community repository (e.g. GitHub). See the Nature Research guidelines for submitting code & software for further information.

Data

Policy information about availability of data

All manuscripts must include a data availability statement. This statement should provide the following information, where applicable:

- Accession codes, unique identifiers, or web links for publicly available datasets
- A list of figures that have associated raw data
- A description of any restrictions on data availability

16s rRNA gene sequence data are available through the NCBI Sequence Read Archive (accession number PRJNA690121). All other data that support the findings of this study are available from the corresponding author upon reasonable request.

Field-specific reporting

Please select the one below that is the best fit for your research. If you are not sure, read the appropriate sections before making your selection.

☒ Life sciences ☐ Behavioural & social sciences ☐ Ecological, evolutionary & environmental sciences

For a reference copy of the document with all sections, see nature.com/documents/nr-reporting-summary-flat.pdf

Life sciences study design

All studies must disclose on these points even when the disclosure is negative.

Sample size	The power calculation for this study was based on previous studies with SHIV and repeat low dose challenges. Resistance to viral acquisition was defined as the number of challenges required for infection (assessed by Mantel Cox test). Modeled Kaplan-Meier curves predicted that an n=10 and a SHIV infection rate of 0.3 per inoculation would detect a 3-fold or greater increase in infection rates in the vaccinated animals relative to the controls. Thus, we enrolled n=10 animals into each experimental group and n=11 animals in the no probiotics/no vaccine control group.
Data exclusions	No data were excluded from the study.
Replication	To date, no replication study has been performed in rhesus macaques to assess the effects of Visbiome(R) probiotics on SIV/HIV DNA/protein vaccination using SIV gag (p55) and HIV env (gp160) plasmids administered by particle mediated epidermal deliver (PMED) and gp140 protein trimer formulated in Adjuplex adjuvant.
Randomization	Male rhesus macaques were randomly assigned to the probiotics+vaccine, vaccine only, probiotics only and no probiotics/no vaccine control groups.
Blinding	Individual experimenters were blinded to animal groups, which was revealed after data collection and analysis were complete.

Reporting for specific materials, systems and methods

We require information from authors about some types of materials, experimental systems and methods used in many studies. Here, indicate whether each material, system or method listed is relevant to your study. If you are not sure if a list item applies to your research, read the appropriate section before selecting a response.

Materials & experimental systems

n/a	Involved in the study	
☐	☒	Antibodies
☒	☐	Eukaryotic cell lines
☒	☐	Palaeontology and archaeology
☐	☒	Animals and other organisms
☒	☐	Human research participants
☒	☐	Clinical data
☒	☐	Dual use research of concern

Methods

n/a	Involved in the study	
☒	☐	ChIP-seq
☐	☒	Flow cytometry
☒	☐	MRI-based neuroimaging

Antibodies

Antibodies used	The use of the following antibodies in flow cytometric analysis are detailed in the manuscript text:<br>CD45-PE (phycoerythrin)-CF594 or -BV786 (D058-1283; BD Biosciences, San Jose, CA); CD3-PerCP (peridinin chlorophyll protein) or BV650 (SP34-2; BD Biosciences); CD4-BV605 (OKT4; BioLegend, San Diego, CA); CD8-APC-H7 (SK1; BD Biosciences) or -BV786 (RPA-T8; BioLegend); CD20-BV570 (2H7; BioLegend); HLA-DR-BV711 (L243; BioLegend); CCR5-PE (3A9; BD Biosciences); CCR6-BV650 (11A9; BD Biosciences); CD28-ECD (CD28.2; Beckman Coulter, Brea, CA); CD95-eFluor450 (DX2; eBioscience/Thermo Fisher Scientific, Waltham, MA); CCR7-FITC (fluorescein isothiocyanate; 3D12; BD Biosciences); IgA-APC (polyclonal; Jackson Immunoresearch, West Grove, PA); IgG-PE-Cy5 (G18-145; BD Biosciences); Ki-67-AF700 (BD56; BD). Sigma-Aldrich); TNF-a-AF700 (eBioscience) or -PE-Cy7 (Mab11; BD Biosciences); IL-17A-PE (ebio64CAP17; eBioscience); IL-22-PerCP-eFluor710 (IL22JOP; eBioscience); IFN-g-BV650 (4S.B3; BioLegend) or -FITC (B27; BD Biosciences); IL-10-PE-Cy7 (JES3-9D7; BioLegend); IL-21-BV421 (3A3-N2.1; BD Biosciences); IL-2-AF700 (MQ1-17H12; BioLegend); CD107a-PE-Cy5 (eBioH4A3; eBioscience); Granzyme B-BV421 (GB11; BD Biosciences)
Validation	All antibodies used for flow cytometry were obtained from commercial vendors that have undergone prior authentication.

Animals and other organisms

Policy information about studies involving animals; ARRIVE guidelines recommended for reporting animal research

Laboratory animals	41 male Indian origin Rhesus macaques (Macaca mulatta)
Wild animals	No wild animals were used in this study.
Field-collected samples	No field-collected samples were used in this study.
Ethics oversight	All animals in this study were housed and cared for at the Washington National Primate Research Center (WaNPRC) under a protocol that was reviewed and approved by the University of Washington Office of Animal Welfare (OWA) Institutional Animal Care and Use Committee (IACUC; Protocol 4266-13; Animal Welfare Assurance Number D16-00292). Animal housing, care and procedures were performed in an AAALAC-accredited facility, in accordance with the regulations put forth by the United States Department of Agriculture, including the Animal Welfare Act (9 CFR) and the Animal Care Policy Manual and with the guidelines established by the National Research Council in the Guide for the Care and Use of Laboratory Animals and the Weatherall Report.

Note that full information on the approval of the study protocol must also be provided in the manuscript.

Flow Cytometry

Plots

Confirm that:

- [x] The axis labels state the marker and fluorochrome used (e.g. CD4-FITC).
- [x] The axis scales are clearly visible. Include numbers along axes only for bottom left plot of group (a 'group' is an analysis of identical markers).
- [x] All plots are contour plots with outliers or pseudocolor plots.
- [x] A numerical value for number of cells or percentage (with statistics) is provided.

Methodology

Sample preparation	Rectum, colon and jejunum biopsies were enzymatically digested and ground through a 70-uM cell strainer into a single cell suspension in R10 medium (RPMI 1640 medium with 2.05nM L-glutamate, supplemented with 10% fetal bovine serum [FBS], 100 U/ml penicillin, and 100ug/ml streptomycin [all from GE Healthcare]). Inguinal or axillary lymph node biopsies were ground through a 70uM cell strainer into a single cell suspension in R10 medium. Peripheral blood mononuclear cells were isolated from whole blood by density gradient centrifugation using Histopaque and Accuspin tubes (both from Sigma-Aldrich). Biopsy and lymph node cell suspensions and PBMCs were stained immediately for flow cytometric analysis.
Instrument	All cytometric acquisition was performed on a BD LSRII cytometer (BD Pharmingen).
Software	Data was collected using FACS Diva software (version 8). Analysis of the acquired data was performed using FlowJo Software (version 9.9.6 or 10.0.8).
Cell population abundance	The abundance of each cell type is presented as a percentage of CD45+ cells, or as a percentage of the parent population (CD4+ T cells, CD8+ T cells, CD20+HLA-DR+ B cells).
Gating strategy	Cells were first identified by excluding doublets using forward scatter (FSC) area and height properties, gating on CD45+ cells, excluding dead cells using an Aqua Live/Dead viability dye and removing any remaining debris using FSC and side scatter (SSC) properties. CD3- cells were identified and B cells were gated as CD20+HLA-DR+ cells. IgA and IgG expressing B cells were identified within CD20+ HLA-DR+ B cells. CD3+ cells were identified and CD4+ and CD8+ T cells were gated with CD3+ cells. Total CD4+ cells were assessed for expression of CCR5, CCR6 and Ki-67. Additionally, within CD4+ T cells, central memory cells were identified by gating on CD28+CD95+ cells and further classified as CCR7+. Effector memory cells were identified as CD28-CD95+ and further classified as CCR7-. Central and effector memory CD4+ T cells were each assessed for expression of CCR5, CCR6 and Ki-67. Total CD8+ cells were assessed for expression of Ki-67. Central memory cells (CD28+CD95+CCR7+) and effector memory cells (CD28-CD95+CCR7-) were identified within CD8+ T cells. Central and effector memory CD8+ T cells were assessed for expression of Ki-67. Cytokine producing cells were identified within CD4+ and CD8+ T cell populations.

- [x] Tick this box to confirm that a figure exemplifying the gating strategy is provided in the Supplementary Information.
